# The advances in creating Crabtree-negative *Saccharomyces cerevisiae* and the application for chemicals biosynthesis

**DOI:** 10.1093/femsyr/foaf014

**Published:** 2025-03-22

**Authors:** Yalin Guo, Zhen Xiong, Haotian Zhai, Yuqi Wang, Qingsheng Qi, Jin Hou

**Affiliations:** State Key Laboratory of Microbial Technology, Shandong University, Binhai Road 72, Qingdao, Shandong, 266237, PR China; State Key Laboratory of Microbial Technology, Shandong University, Binhai Road 72, Qingdao, Shandong, 266237, PR China; State Key Laboratory of Microbial Technology, Shandong University, Binhai Road 72, Qingdao, Shandong, 266237, PR China; State Key Laboratory of Microbial Technology, Shandong University, Binhai Road 72, Qingdao, Shandong, 266237, PR China; State Key Laboratory of Microbial Technology, Shandong University, Binhai Road 72, Qingdao, Shandong, 266237, PR China; State Key Laboratory of Microbial Technology, Shandong University, Binhai Road 72, Qingdao, Shandong, 266237, PR China

**Keywords:** *Saccharomyces cerevisiae*, Crabtree effect, pyruvate decarboxylase deficient strains, metabolic engineering, ethanol

## Abstract

*Saccharomyces cerevisiae* is a promising microbial cell factory. However, the overflow metabolism, known as the Crabtree effect, directs the majority of the carbon source toward ethanol production, in many cases, resulting in low yields of other target chemicals and byproducts accumulation. To construct Crabtree-negative *S. cerevisiae*, the deletion of pyruvate decarboxylases and/or ethanol dehydrogenases is required. However, these modifications compromises the growth of the strains on glucose. This review discusses the metabolic engineering approaches used to eliminate ethanol production, the efforts to alleviate growth defect of Crabtree-negative strains, and the underlying mechanisms of the growth rescue. In addition, it summarizes the applications of Crabtree-negative *S. cerevisiae* in the synthesis of various chemicals such as lactic acid, 2,3-butanediol, malic acid, succinic acid, isobutanol, and others.

## Teaser

*Saccharomyces cerevisiae* is a Crabtree-positive yeast and accumulates ethanol as main product. This review discusses the efforts to create Crabtree-negative stains and the applications to produce chemicals beyond ethanol.

## Introduction

*Saccharomyces cerevisiae* is widely recognized as a robust ethanol producer, and recent efforts have increasingly focused on engineering it for cellulosic ethanol production (Hou et al. 2017, Sharma et al. 2022). Aside from ethanol, it has also been widely used as a microbial cell factory for producing fuels (Nielsen et al. 2013), chemicals (Wang et al. 2024), food ingredients (Pretorius 2017), and pharmaceuticals (Nielsen 2019). *Saccharomyces cerevisiae* has significant advantages as a microorganism cell factory. It has a clear genetic manipulation background, a strong capacity for homologous recombination (Mathiasen and Lisby 2014). Numerous genetic engineering tools have been developed (Guirimand et al. 2021, Zhai et al. 2022). Furthermore, it has been used in food production for a long time and is recognized as a Generally Recognized As Safe organism by the US Food and Drug Administration. It is a preferred cell factory, particularly for the production of natural products that require the expression of eukaryotic-derived enzymes (Nett 2024). In addition, its high tolerance to stressors such as low pH makes it suitable for producing organic acids (Nevoigt 2008).

One of the important metabolic features of *S. cerevisiae* is the Crabtree effect. That is, under aerobic and anaerobic conditions, it does not rely on oxidative phosphorylation to produce adenosine triphosphate (ATP), but rather through phosphorylation at the substrate level. When the concentration of glucose in the environment is high, respiration of *S. cerevisiae* is inhibited and glucose rapidly produces ethanol through fermentation (Hagman and Piškur 2015). In this process, a high glycolytic flux meets the energy demands of rapid cell growth, and the ethanol released can inhibit the growth of competitors, giving *S. cerevisiae* an advantage in natural evolution (Rozpędowska et al. 2011). Subsequently, under aerobic conditions, ethanol is utilized as both an energy and carbon source in a metabolic process known as “diauxic shift” (Pronk et al. 1996). However, the use of respiration is much more efficient than fermentation, and respiration produces ~10 times more ATP from the full oxidation of glucose than fermentation does (Pfeiffer and Morley 2014, Nilsson and Nielsen 2016, Malina et al. 2021).

Many efforts have been made to explain the Crabtree effect of *S. cerevisiae* (Piskur et al. 2006). One hypothesis is the limitation of respiratory capacity, meaning that the mitochondrial electron transport chain of *S. cerevisiae* is not able to efficiently oxidize nicotinamide adenine dinucleotide (NADH) (Aceituno et al. 2012). This results in excessive NADH accumulation, which needs to be consumed via ethanol production. This hypothesis is supported by several experimental findings, such as a significant reduction in metabolic overflow to ethanol when expressing a heterologous alternative oxidase (Vemuri et al. 2007). Similarly, when the oxidase HaAOX1 from *Hansenula anomala* was expressed in yeast, respiratory chain complex III was upregulated, and tricarboxylic acid (TCA) cycle activity increased (Mathy et al. 2006). In addition, a new perspective explains this phenomenon in terms of ATP production and the cost of metabolism and protein translation (Shen et al. 2024). Chen et al. estimated substrate and protein costs for synthesizing metabolites based on genome-scale metabolic modeling in conjunction with amino acid abundance (Chen and Nielsen 2022). The results suggest that *S. cerevisiae* tends to utilize its resources in the pathway with the lowest cost of protein synthesis, rather than aiming to produce more ATP through respiration, as respiration requires more proteins (Chen and Nielsen 2022). In addition, under respiro-fermentative conditions, the translation efficiency is lower than respiratory metabolism state, which also leads to a decrease in protein levels (Gancedo 2008, Malina et al. 2021). Overall, although Crabtree-positive yeasts are quantitatively inefficient at producing ATP via the fermentation pathway, faster glucose uptake rate and higher glycolytic flux compensate for the inefficiency, and fermentation minimizes the cost of protein synthesis (Metzl-Raz et al. 2017, Chen and Nielsen 2019).

The Crabtree effect is not present in all yeasts. There are also Crabtree-negative yeasts, such as *Kluyveromyces marxianus* and *Scheffersomyces stipitis*. They undergo complete oxidation through glycolysis, the tricarboxylic acid cycle, and the respiratory chain to produce ATP (Malina et al. 2021). As a Crabtree-positive yeast, *S. cerevisiae* rapidly transfers a large carbon flux to ethanol in the natural environment, which is subsequently used for growth and biomass generation (Qi et al. 2024). This facilitates industrial fermentation of ethanol, but is not favorable for using yeast to the production of other chemicals. Therefore, metabolic engineering using *S. cerevisiae* to produce other chemicals often requires blocking or reducing ethanol production, i.e. disrupting the Crabtree effect.

## Blocking the production of ethanol in *S. cerevisiae* to eliminate the Crabtree effect

**Figure 1.**
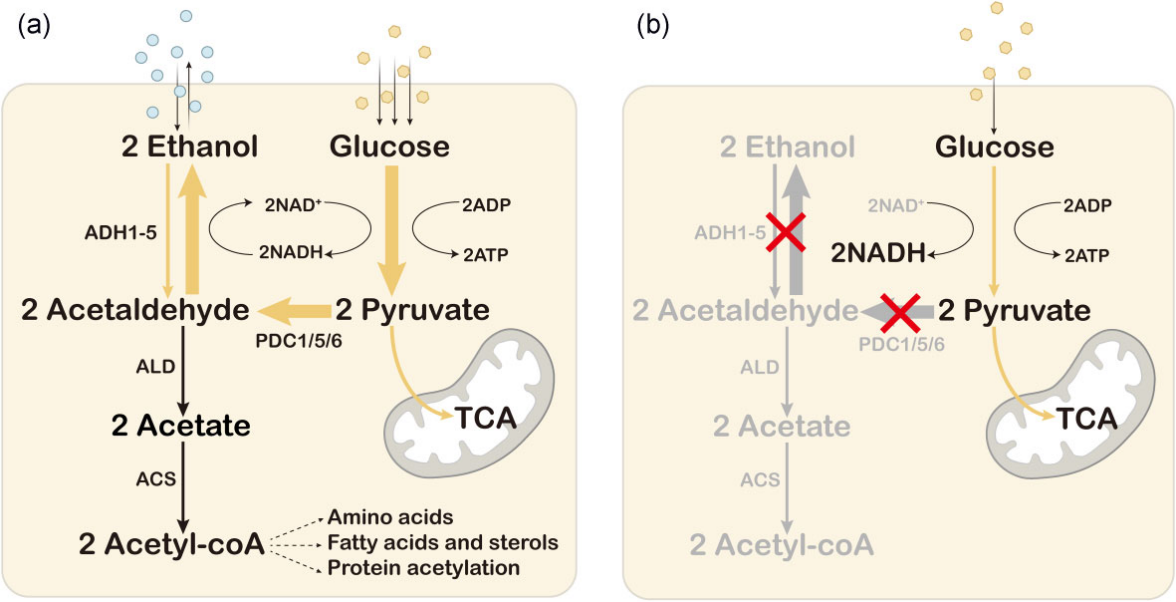
Comparison of wild *S. cerevisiae* (Crabtree-positive) and Crabtree-negative *S. cerevisiae* for eliminating ethanol accumulation. (a) Wild-type *S. cerevisiae* rapidly takes up glucose. Glucose is consumed through glycolysis, releasing large amounts of NADH, which is oxidized to produce ethanol. This process involves phosphorylation at the substrate level. Ethanol then serves as a carbon source and the strain depends on mitochondria for secondary growth through respiration. (b) *Saccharomyces cerevisiae* that eliminates ethanol production is considered Crabtree-negative. *ADH* deletion results in intracellular cofactor imbalance due to inability to oxidize NADH. *PDC* deletion not only causes an intracellular cofactor imbalance but also results in cytoplasmic acetyl-CoA deficiency. Thus, cell growth is impaired. The intracellular metabolic burden is high, and energy can only be generated through impaired oxidative phosphorylation. *PDC*, pyruvate decarboxylase; *ADH*, ethanol dehydrogenase; *ALD*, acetaldehyde dehydrogenase; and *ACS*, acetyl-CoA synthetase.

Ethanol is a key metabolite of *S. cerevisiae*. It is a metabolite of the glycolytic and fermentative pathways and a substrate for aerobic respiration (Fig. 1). After glucose enters the cell, ethanol is produced through glycolysis and pyruvate dehydrogenase (Pdh) bypass. First, one molecule of glucose produces two molecules of pyruvate, two molecules of NADH, and two molecules of ATP through glycolysis. Pyruvate then enters the Pdh bypass. Pyruvate decarboxylase (Pdc) catalyzes the production of acetaldehyde and CO_2_ from pyruvate, and acetaldehyde is catalyzed by ethanol dehydrogenase (Adh) to ethanol while oxidizing NADH to NAD^+^. Meanwhile, acetaldehyde is catalyzed by acetaldehyde dehydrogenase (Ald) to produce acetic acid while consuming NAD(P)^+^, and acetic acid is catalyzed by acetyl coenzyme A synthetase (Acs) to produce acetyl coenzyme A, which is an important source of lipid synthesis (Dai et al. 2018). To increase the flux of metabolic pathways that generate the target product, elimination of byproduct production is essential. It is difficult to completely eliminate ethanol production in *S. cerevisiae*. Therefore, attenuating its Pdh bypass, i.e. combinatorial deletions of *PDC* and/or *ADH*, is a common strategy for attenuating ethanol production and Crabtree effect.

### The deletion of *PDC* isoenzymes

In *S. cerevisiae*, Pdc has very high activity, which is one of the bases of the Crabtree effect (Pronk et al. 1996, Agarwal et al. 2013). Pdc1, Pdc5, and Pdc6 are three isoenzymes. Pdc1 plays a major role and has a very high expression level. Pdc5 is only active when there is no Pdc1 protein expression, because its promoter is inhibited by Pdc1 protein (Eberhardt et al. 1999). Pdc2 is not a direct *PDC* gene, but a transcription factor (Iosue et al. 2023). It regulates the expression of Pdc1 and Pdc5, and is important for their functions (Mojzita and Hohmann 2006b, Nosaka et al. 2012). With a nonfermentable carbon source, Pdc6 is activated under sulfur limitation. When glucose is the carbon source, *PDC6* cannot be transcribed (Boer et al. 2003).

Combinatorial deletion of Pdc isozymes has different effects in attenuating ethanol production. After knocking out *PDC1*, the strains PS3-1-5b and PS3-2-3b can grow on glucose and still have most of the Pdc activity (Seeboth et al. 1990). When glucose was used as the carbon source, the knockout of *PDC1* and *PDC5* in a lactic acid-producing strain LA1 resulted in a 1.9-fold increase in lactic acid (LA) production and a 1.8-fold reduction in ethanol flux (Pangestu et al. 2022). Zhang et al. (2022a) also observed a reduction in ethanol synthesis a partial reduction, with ethanol conversion decreasing by 30.19% and Pdc activity dropping by 40.91% in strain H14-02 following the knockout of *PDC1/5*. Deletion of *PDC2* resulted in a significant decrease in the expression level of *PDC1* and an almost undetectable level of Pdc5 (Mojzita and Hohmann 2006a, Iosue et al. 2023). However, the exact mechanism of *PDC2* remains unclear. When a truncated Pdc2^△519^ was used, ethanol production in BY4743 was reduced by 7.4%, while cell growth was barely affected (Cuello et al. 2017). Deletion of all *PDC* genes prevented ethanol production, but because the Pdc defect resulted in a deficiency of cytoplasmic acetyl-coenzyme A, the strain could not grow in medium with glucose as the sole carbon source (Flikweert et al. 1996, Zhang et al. 2015b) (Fig. 1). Growth can be partially restored by adding acetic acid or ethanol to the medium (Van Maris et al. 2004a).

### The deletion of ADH isoenzymes

Adhs are enzymes for the final step of ethanol production. They reduce acetaldehyde to ethanol, regenerating NAD^+^ required for glycolysis (Fig. 1). Some Adhs also oxidize ethanol to acetaldehyde, preparing the cell for the next step of using aerobic respiration to break down ethanol. They link fermentation and respiration to optimize cellular carbon utilization. Reducing ethanol production by deleting *ADH* avoids cytoplasmic acetyl-CoA deficiency. In *S. cerevisiae*, there are five classical *ADHs* (*ADH1/2/3/4/5*), the isoenzyme Sfa1, and Adh6/7 (Dickinson et al. 2003, De Smidt et al. 2008, 2012). Among them, Adh1 is the main enzyme that catalyzes the reduction of acetaldehyde and is a key enzyme in NADH oxidation during aerobic fermentation (Jacobi et al. 2024).

Combinatorial deletions of Adh isozymes also show different effects in attenuating ethanol production. When only Adh1 was expressed and other isozymes were absent, the strain grew similarly to the parental strain. It could produce ethanol in aerobic fermentation and slowly oxidize it during ethanol recovery (Wills 1976). Deletion of *ADH1* resulted in weakened ethanol synthesis, and toxic effects from acetaldehyde accumulation, leading to slow growth on glucose. However, the strain could adjust its metabolic pattern and recover with the help of other isoenzymes such as Adh2 and Adh4 (Ida et al. 2012). Mutants with a double deletion of *ADH1* and *ADH2* showed slower growth on glucose but were still able to produce ethanol (Kusano et al. 1998). Deletion of *ADH1, ADH2, ADH3, ADH4, ADH5*, and *SFA1* in BY4739 eliminated ethanol accumulation and shifted the strain to accumulate glycerol, but growth is significantly affected (Ida et al. 2012).

## Rescue growth defects of Crabtree-negative strains

When glucose is used as a carbon source, *S. cerevisiae* produces energy by fermentation and respiration. With high glucose concentrations, *S. cerevisiae* tends to ferment metabolism, i.e. to produce ethanol, even when oxygen is abundant. When producing other metabolites, ethanol accumulation is not desired. Therefore, to remove ethanol production, *PDC1, PDC5*, and *PDC6* were often deleted to create Crabtree-negative strains, but Crabtree-negative strains have growth defect (Oud et al. 2012). It is suspected that the deficiency of intracellular C2 compounds and cofactor imbalance are the possible causes of growth defect. First, *PDC* deletion causes a deficiency of intracellular C2 compounds, such as ethanol and acetic acid, resulting in a lack of acetyl-CoA in the cytoplasm. Thus, the synthesis of lysine and fatty acids is affected, which is essential for cell growth (Flikweert et al. 1999, Pham et al. 2022). Second, Crabtree-negative strains grow very slowly on fermentable carbon sources because of the redox imbalance. NADH produced by glycolysis is generally oxidized by Adh to generate ethanol. The deletion of *PDC* blocks this reaction, meanwhile glucose inhibits respiration and NADH cannot be fully oxidized through the respiratory chain, thereby cause cofactor imbalance (Pronk et al. 1996). Researchers have applied adaptive laboratory evolution (ALE) and cytoplasmic acetyl-CoA supplementation to restore the growth defect (Wang et al. 2012). ALE combined with reverse engineering, has led to the discovery of key targets related to Crabtree effect (Van Maris et al. 2004a). These studies provide new insights for the study and application of Crabtree-negative yeasts.

### ALE and identify the genes that can rescue the growth defect

**Figure 2.**
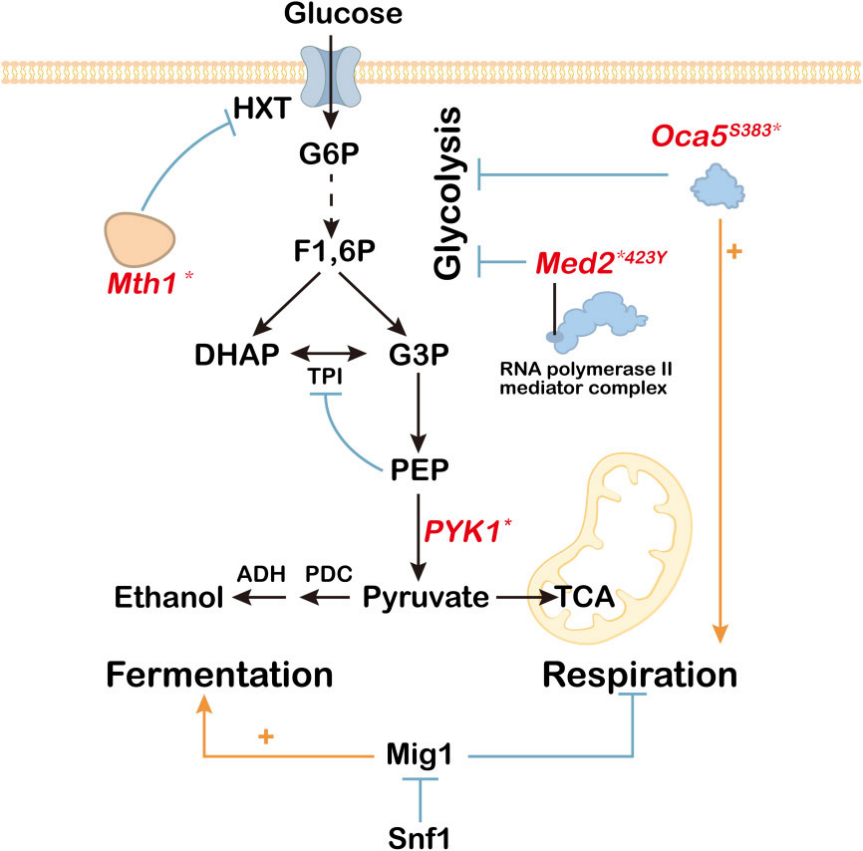
The key genes identified in ALE that can rescue the growth defect. Mth1* includes Mth1^∆57–131^, Mth1^A81D^, and Mth1^A81P^, which is described in more detail in Fig. 3. PYK1* includes PYK1^R68*^, PYK1^K196*^, and *PYK1^R91I^*. Mutations in Pyk1 causes the accumulation of PEP and downregulates glycolysis. Med2 is a component of the tail module of the RNA polymerase II mediator complex, which affects the transcriptional regulation of genes dependent on RNA polymerase II. The MED2^*432Y^ mutation impacts global metabolic networks, leading to downregulation of glycolysis and upregulation of genes involved in protein synthesis. The Snf1/Mig1 pathway regulates gluconeogenesis, respiration, and sugar transport. Deletion of *SNF1* and *MIG1* results in increased respiration and decreased overflow metabolism. Oca5 is an inositol pyrophosphatase. OCA5^S383*^ or deletion of *OCA5* inhibits fermentation and improves respiration.

Growth defects in *S. cerevisiae* strains caused by the absence of *PDC* can be rescued by ALE. Due to the lack of cytoplasmic C2 compounds, the initial evolution medium is often supplemented with moderate amounts of acetic acid or ethanol, and the concentration of C2 compounds is gradually reduced in subsequent transitions (Van Maris et al. 2004a, Oud et al. 2012). In order to release the inhibition of the strain by glucose, the glucose concentration in the medium was gradually increased in the following evolutions. The strains with improved growth rate were isolated, and combining whole genome sequencing and reverse engineering, a number of key gene targets for restoring the growth of PDC-negative strains were identified (Fig. 2). For example, the mutations of Mth1 including Mth1^∆57–131^ (Oud et al. 2012), Mth1^A81D^ (Zhang et al. 2015b), and Mth1^A81P^ (Kim et al. 2013b) were found to restore the cell growth. Mth1 mutations are primarily associated with glucose uptake. Pyruvate kinase mutations Pyk1^R68*^, Pyk1^K196*^, Pyk1^R91I^ (Yu et al. 2018), pyrophosphatase Oca5^S383*^ (Qin et al. 2023), and RNA polymerase II mediator complex Med2^*432Y^ (Dai et al. 2018) were also identified which have a beneficial effect on balancing glycolysis and respiratory metabolism.

#### Engineering signaling pathways to reduce glucose uptake

PDC-negative strains have impaired ethanol synthesis and are unable to rapidly consume NADH (Jouandot et al. 2011), resulting in an intracellular cofactor imbalance. Limiting glucose utilization can reduce NADH production and restore intracellular NAD^+^/NADH balance.

**Figure 3.**
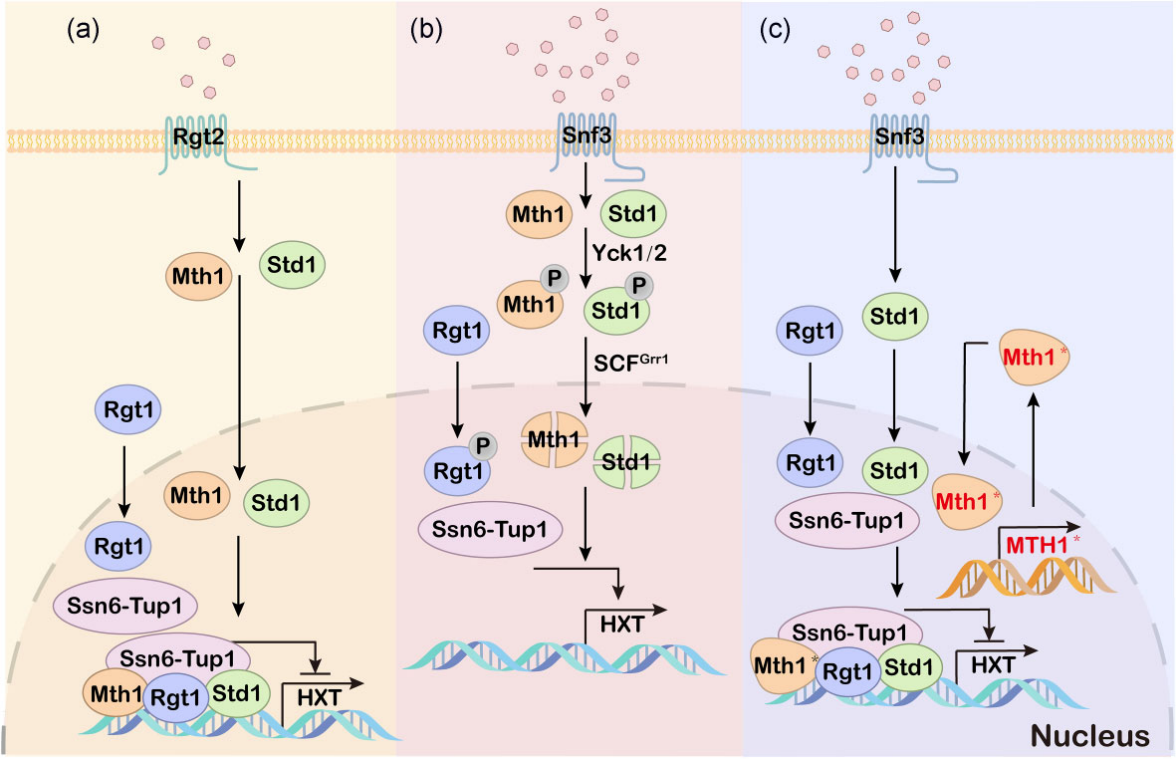
Effect of *MTH* mutations on glucose uptake. Mth1* includes Mth1^∆57–131^, Mth1^A81D^, and Mth1^A81P^. (a) Signal transduction pathway at low glucose concentration. Rgt2 senses low glucose concentration. Rgt1 recruits Mth1, Std1, and Ssn6–Tup1 to the *HXT* promoter to represse *HXT* expression. (b) Signal transduction pathway at high glucose concentration. Snf3 senses high glucose concentration. Casein kinase Yck1 and Yck2 phosphorylate Mth1 and Std1, and Mth1 and Std1 are consequently degraded. Rgt1 dissociates from Ssn6–Tup1. *HXT* is expressed and glucose uptake is accelerated. (c) Signal transduction pathway after mutation of Mth1 at high glucose concentration. Deletion of the phosphorylation site in mutant Mth1 results in enhanced stability or delays degradation by structural changes in the protein. *HXT* expression is inhibited and the glucose uptake rate of mutant strain does not increase with increasing extracellular glucose concentration. The lower glucose uptake rate also mitigates the Crabtree effect.

Glucose uptake is the rate-limiting step in glucose utilization. *Saccharomyces cerevisiae* has complex signaling pathways to sense glucose and regulate its uptake and metabolism (Roy et al. 2013, Conrad et al. 2014) (Fig. 3). These include the Snf3/Rgt2-Rgt1, also known as sensor/receptor–repressor glucose signaling pathway (Snowdon and Johnston 2016, Qiu et al. 2023). The pathway begins with the glucose-sensing receptors (GSRs) Rgt2 and Snf3 on the cell membrane and ends with transcriptional regulation of the *HXT* genes by the transcriptional repressor Rgt1 in the nucleus (Kim et al. 2022). Snf3 and Rgt2 detect low and high concentrations of extracellular glucose, respectively (Kayikci and Nielsen 2015, Kim and Rodriguez 2021). In the absence of glucose, Rgt1 recruits HXT corepressors Mth1 and Std1. It also recruits the universal corepressor complex Ssn6–Tup1 to the HXT promoter. This leads to repression of HXT gene expression (Kim et al. 2022). In this process, Mth1 can prevent the phosphorylation of Rgt1, which binds to DNA to achieve transcriptional repression (Polish et al. 2005). When extracellular glucose concentrations are elevated, the GSR conformation changes. The casein kinases Yck1 and Yck2, which are anchored to the plasma membrane, phosphorylate Mth1 and Std1 (Roy et al. 2016). Then Scf^Grr1^ ubiquitin-protein ligase recognizes and degrades Mth1 and Std1, leading to the dissociation of Ssn6–Tup1 from Rgt1. It in turn leads to the expression of *HXT* genes (Moriya and Johnston 2004).

ALE of PDC-negative strains has identified a number of effective mutations encoding *MTH1*, a negative regulator of the glucose sensing signaling pathway. For example, a deletion of 225 bp in the *MTH1* gene (corresponding to amino acids 57–131), which contains the phosphorylation site required for degradation, leads to enhanced stability of the Mth1 protein (Oud et al. 2012). This is because only after Mth1 is phosphorylated by Yck1, its ubiquitination by SCF^Grr1^ is triggered and subsequently degraded by the proteasome. The mutation causes the reduction in Mth1 degradation and slows down glucose uptake (Moriya and Johnston 2004). The deletion of this 225 bp in *MTH1* in Crabtree-negative strains resulted in a specific growth rate up to 0.097 h^−1^ when grown on medium with glucose as the sole carbon source, suggesting that the introduction of this mutation enables PDC-negative strains to grow in the medium with glucose as the sole carbon source (Oud et al. 2012). In another study, a mutation in *MTH1* allele Bpc1-1^I85N^ and a commutation in alleles Dgt1-1^I85N^ and Dgt1-1^S102G^ were identified, which contribute to reducing glucose transport and alleviate catabolite repression (Lafuente et al. 2000). Introduction of the Mth1^A81D^ allele into an unevolved strain resulted in a maximum specific growth rate of 0.053 h^−1^ in minimal medium containing 2% glucose (Zhang et al. 2015b). This mutation affects the function of the helical structure within the conserved island of Mth1^71–91^, and thus exhibits a similar function in alleviating glucose repression. Sequencing analysis of an evolved strain defective in Pdc for the production of 2,3-butanediol (2,3-BDO) revealed a polynucleotide polymorphism (SNP) at position 231, which resulted in Mth1^A81P^. The mutation from alanine to proline may alter protein structure that delay the degradation of Mth1, so the rate of glucose uptake in the mutant strain does not increase with increased external glucose concentrations (Kim et al. 2013a).

#### Balancing glycolysis and respiratory metabolism

*Saccharomyces cerevisiae* grows with a high glycolytic flux and produces large amounts of NADH. In *PDC*-negative strains, ethanol production is blocked and NAD^+^ cannot be regenerated. On the other hand, the Crabtree effect inhibits the respiratory chain and NADH cannot be oxidized efficiently by oxidative phosphorylation (Bakker et al. 2001). This leads to an intracellular NADH/NAD^+^ imbalance, i.e. redox homeostasis cannot be maintained. The intracellular redox homeostasis can be restored by limiting glycolytic flux and relieving respiratory inhibition (Fig. 2).

Pyruvate kinase (Pyk) is a key control point for glycolytic flux and catalyzes the conversion of phosphoenolpyruvate (Pep) and adenosine diphosphate (ADP) to pyruvate and ATP. Yu et al. (2018) performed whole-genome sequencing of three separate clones isolated in the ALE experiment and found that they had mutations in Pyk1^R68*^, Pyk1^K196*^, and Pyk1^R91I^, respectively, and the evolved strains had much lower total Pyk activity than the unevolved strains, but elevated Pyk2 activity. Pyk1 is predominant Pyk and tightly regulated by fructose 1,6-bisphosphate (Fbp), whereas the isoenzyme Pyk2 is insensitive to Fbp but is inhibited by glucose (Chen et al. 2021). When Pyk activity is low, its substrate Pep accumulates, and Pep is a feedback inhibitor of triosephosphate isomerase, so the downregulation of Pyk1 reduces glycolysis. In addition, oxygen consumption and respiratory activity can be increased when Pyk activity is reduced (Grüning et al. 2011). To restore the growth of the PDC-negative strain, Dai et al. (2018) introduced a non-ATP-dependent cytoplasmic acetyl-CoA production pathway and performed ALE for 40 days. Med2^*432Y^ was identified in subsequent genome sequencing and reverse engineering. The introduction of Med2^*432Y^ increased the specific growth rate by 47%, compared to the control strain to 0.156 h^−1^. Med2 is a component of the tail module of the RNA polymerase II mediator complex (Van de Peppel et al. 2005). It affects the transcriptional regulation of the genes dependent on RNA polymerase II (Dotson et al. 2000). The results of transcriptome analysis showed that the mutation in Med2 had an impact on the global metabolic network, where the genes related to carbon metabolism, such as glycolysis were downregulated. The genes related to protein synthesis were upregulated, which explains the increased rate of cellular growth. Zhang et al. (2022b) introduced the Med2^*432Y^ mutation into a wild-type strain and found that biomass increased and the rate of sugar consumption was decreased, suggesting that the mutation improved the energy efficiency of cellular metabolism. Further introduction of Mth1^A81D^ to this strain resulted in faster cell growth and less ethanol accumulation (Zhang et al. 2022b).

PDC-negative strains slow down glucose utilization, and the rate of product production decreases. Therefore, alleviation of glucose inhibition and enhancement of respiratory metabolism are of high value in industrial applications. Van Maris et al. (2004a) identified the downregulation of the transcriptional repressor Mig1 in an ALE strain. Often mentioned along with Mig1 is the protein kinase Snf1. Snf1 interacts with many transcription factors that regulate gluconeogenesis, respiration, and *HXT* expression (Kayikci and Nielsen 2015), and one of its major targets is Mig1 (Persson et al. 2022). Deletion of snf1 and its target Mig1 results in increased respiration and decreased overflow metabolism (Moriya and Johnston 2004, Baek et al. 2016a). Furthermore, *SNF1* deletion increased cellular mitochondrial respiration at a 10% glucose concentration, suggesting that *SNF1* is involved in the transition between mitochondrial respiration and fermentation (Martinez‐Ortiz et al. 2019). Hexose phosphate during glycolysis has an important influence on respiratory activity or the occurrence of the Crabtree effect. F1,6bP inhibits mitochondrial complexes III and IV, while G6P has an activating effect on the respiratory chain (Díaz-Ruiz et al. 2008). It was later found that the ratio of G6P to F1,6bP must be below 0.7–0.8 to trigger the Crabtree effect. Conversely, when the ratio is greater than 0.8, it increases cellular respiration (Rosas Lemus et al. 2018). Therefore, regulating the ratio of G6P and F1,6-BP in cells can enhance cellular respiration. Qin et al. identified the Oca5^S383*^ mutation following adaptive evolution of a hybrid glycolytic yeast. Reverse engineering showed that the mutation restored growth in the unevolved strain, and when the entire open reading frame of Oca5 was deleted, the maximum specific growth rate of the mutant strain was even higher than that of the evolved strain. Oca5 is an inositol pyrophosphatase, and its deletion inhibits fermentation and improving respiration (Qin et al. 2023). Although the mutations identified from the evolved strains are different, they generally play a role in limiting glucose uptake, decreasing glycolytic flux, and elevating respiratory metabolism (Fig. 2). These mutations balance the flux of glycolysis, TCA cycle, and oxidative phosphorylation, thereby contributing to NAD^+^/NADH homeostasis and growth recovery.

### Increasing acetyl-CoA supply

**Figure 4.**
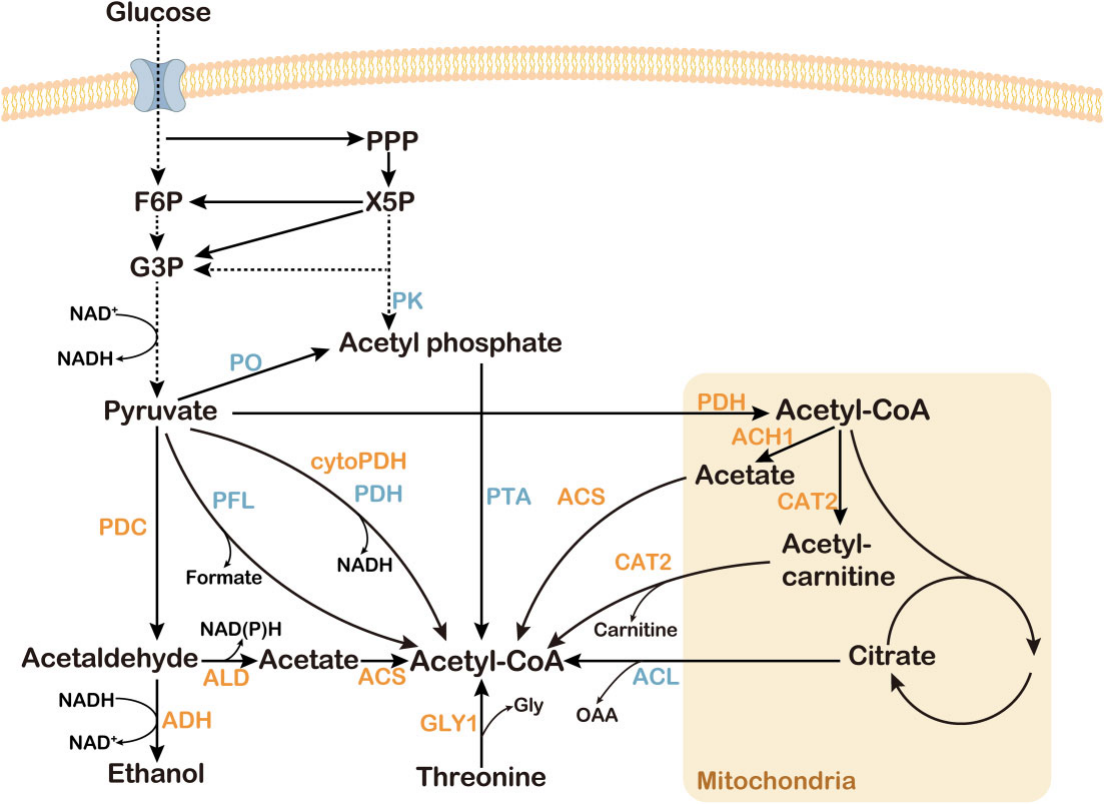
Strategies for supplementation of cytoplasmic acetyl-CoA. *Saccharomyces cerevisiae* produces acetyl-CoA mainly through the mitochondrial pathway and the Pdh bypass. In the mitochondria, pyruvate is converted by Pdh into acetyl-CoA. In the cytoplasm, pyruvate is catalyzed by Pdc, Ald, and acetyl-CoA synthetase (Acs) in the Pdh bypass to yield acetyl-CoA. Acetaldehyde is also catalyzed by Adh to ethanol while oxidizing NADH to NAD^+^. Other endogenous enzymes that generate acetyl-CoA include threonine aldolase (Gly1), mitochondrial CoA transferase (Ach1), removal of the Pdh MTS to generate cytoplasmic Pdh (cytoPdh), and carnitine acetyltransferase (Cat2). Heterologous enzymes introduced into *S. cerevisiae* to replenish acetyl-CoA include pyruvate-formate lyase (Pfl), pyruvate oxidase (Po), or phosphoketolase (Pk) with phosphotransacetylase (Pta), derived from bacterial Pdh, ATP-dependent citrate lyase (Acl).

Acetyl-CoA is essential for cell growth and is an important precursor for the synthesis of amino acids, fatty acids, sterols, and other compounds (Kanehisa et al. 2014). In *S. cerevisiae*, acetyl-CoA is produced in the nucleus, mitochondria, cytoplasm, and peroxisome, but is not capable of direct transport between organelles (Krivoruchko et al. 2015). Thus, the deletion of the three *PDC* genes, *PDC1, PDC5*, and *PDC6*, does not lead to ethanol accumulation, but prevents growth on glucose because of the lack of cytoplasmic acetyl-CoA. Supplemented with C2 compounds such as ethanol and acetic acid, cytoplasmic acetyl-CoA can be replenished (Van Maris et al. 2003, Lian et al. 2014a). In addition, cytoplasmic acetyl-CoA can also be supplemented by overexpressing or introducing key enzymes (Fig. 4).

Many strategies to replenish cytoplasmic acetyl-CoA have been applied to PDC-negative strains. Threonine aldolase cleaves threonine into glycine and acetaldehyde, and acetaldehyde can be used as a precursor for acetyl-CoA (Monschau et al. 1997). Van et al. overexpressed *GLY1* in PDC-negative yeast and found that growth with glucose as the sole carbon source was partially restored, suggesting that Gly1 catalyzes the production of acetaldehyde to complement cytoplasmic acetyl-CoA (Van Maris et al. 2003). PDC-negative strains accumulate large amounts of pyruvate, which serves as a precursor for acetyl-CoA. Pyruvate-formate lyase (Pfl) converts pyruvate to formate and acetyl-CoA. Zhang et al. (2015a) expressed Pfl from *Escherichia coli* in a PDC-negative *S. cerevisiae*, and coexpression of the combined electron donor had a positive effect on yeast growth under aerobic conditions (Zhang et al. 2015a). Pfl could restore growth of *ACS1*- and *ACS2*-deficient strains on glucose. Formate is toxic, and its reoxidation should be considered (Kozak et al. 2014a). Zhang et al. (2015b) performed an ALE on PDC-negative strains. The evolved strains were found to have point mutations in mitochondrial citrate synthase, which exhibited lower Cit1 activity. Deletion of *CIT1* increased the maximum specific growth rate from 0.053 to 0.069 h^−1^ in glucose-containing medium (Zhang et al. 2015b). Cit1 catalyzes the formation of citric acid by the condensation of acetyl-CoA and oxaloacetate. Mitochondrial CoA transferase (Ach1) catalyzes the conversion of acetyl-CoA to acetic acid (Chen et al. 2015). The absence of Cit1 reduces competition for acetyl-CoA. Thus, mitochondrial Ach1 can convert more acetyl-CoA into acetic acid, which crosses the mitochondrial membrane to replenish cytoplasmic acetyl-CoA (Chen et al. 2015). Dai et al. (2018) introduced the PO/PTA pathway into a PDC-negative strain to construct an ATP-independent pathway for acetyl-CoA production. This pathway includes pyruvate oxidase (Po) from *Aerococcus viridans*, which catalyzes the decarboxylation of pyruvate to acetyl phosphate, phosphotransacetylase (Pta) from *Salmonella enterica*, which catalyzes the conversion of acetyl phosphate to acetyl-CoA. When Pta and Po replaced Acs1 and Acs2, the derivatives of acetyl-CoA, 3-hydroxypropionate (3-HP) and farnesene, accumulated to 20.5 mg l^−1^ and 61.4 mg l^−1^, respectively, demonstrating that this pathway effectively supplements the cytoplasm acetyl-CoA of PDC-negative *S. cerevisiae*.

A number of other strategies to supplement cytoplasmic acetyl-CoA have yet to be applied to PDC-negative strains. Experimental results also showed the potential of these strategies. Nonoxidative glycolysis (NOG), which refers to the combination of glycolysis, the pentose phosphate pathway, and phosphoketolase (Pk) and Pta have been described previously (Bogorad et al. 2013). The xylulose-5-phosphate-specific phosphoketolase (Xpk) from *Leuconostoc mesenteroides*, which catalyzes the cleavage of xylulose-5-phosphate to acetylphosphate and glyceraldehyde-3-phosphate, and the Pta from *Clostridium krusei* were used to enhance 3-HP production (Qin et al. 2020). NOG converts glucose to acetyl-CoA without CO_2_ emissions. Therefore it is considered as a carbon conservation pathway. Similarly, in another study, heterologous expression of these two enzymes and a bacterial transhydrogenase (which catalyzes the production of NADH from NADPH) rescued the growth of a glycolysis-deficient strain (Qin et al. 2023). Pdh is a three-subunit complex that catalyzes the generation of acetyl-CoA from pyruvate at a low energy cost (Kozak et al. 2014b). Lian et al. (2014b) removed the MTS of *S. cerevisiae* Pdh to obtain cytoPDH to complement cytoplasmic acetyl-CoA. Lian et al. (2014b) also explored the expression of *E. coli*-derived Pdh in *S. cerevisiae*, which does not possess MTS and allows direct cytoplasmic localization. Similarly, Zhang et al. (2020) showed that Pdh from *Enterococcus faecalis* could completely replace the endogenous cytoplasmic acetyl-CoA synthesis pathway in *S. cerevisiae*. In mitochondria, the carnitine acetyltransferase Cat2 catalyzes the formation of acetyl-CoA and carnitine to form acetyl-carnitine, allowing acetyl-carnitine enters the cytosol to replenish cytoplasmic acetyl-CoA (Van Roermund et al. 1999, Franken et al. 2008). Van Rossum et al. (2016) obtained yeast strains dependent on the carnitine shuttle system for cytoplasmic acetyl-CoA replenishment after deleting the *PDH* complex and performing adaptive evolution. The citrate-oxaloacetate shuttle system transfers citric acid from the mitochondria to the cytoplasm, where ATP-dependent citrate lyase (Acl) cleaves citric acid and forms acetyl-CoA and oxaloacetate with coenzyme A (Verschueren et al. 2019). It has been studied to introduce heterologous Acl to increase yeast acetyl-CoA, thereby increasing the yield of target compounds (Lian et al. 2014b, Rodriguez et al. 2016, Zhang et al. 2020). The replenishment of cytoplasmic acetyl-CoA not only supplies C2 compounds in PDC-negative strains, but also provides precursors for biosynthesis. The cytoplasmic acetyl-CoA can be used to synthesize a variety of chemicals such as free fatty acids and 3-hydroxypropionic acid. When selecting the strategies for cytoplasmic acetyl-CoA supplementation, the compartmentalized distribution of metabolic pathways, the carbon yield, and the consumption of energy should be considered.

## Application of Crabtree-negative strains as microbial cell factories

**Figure 5.**
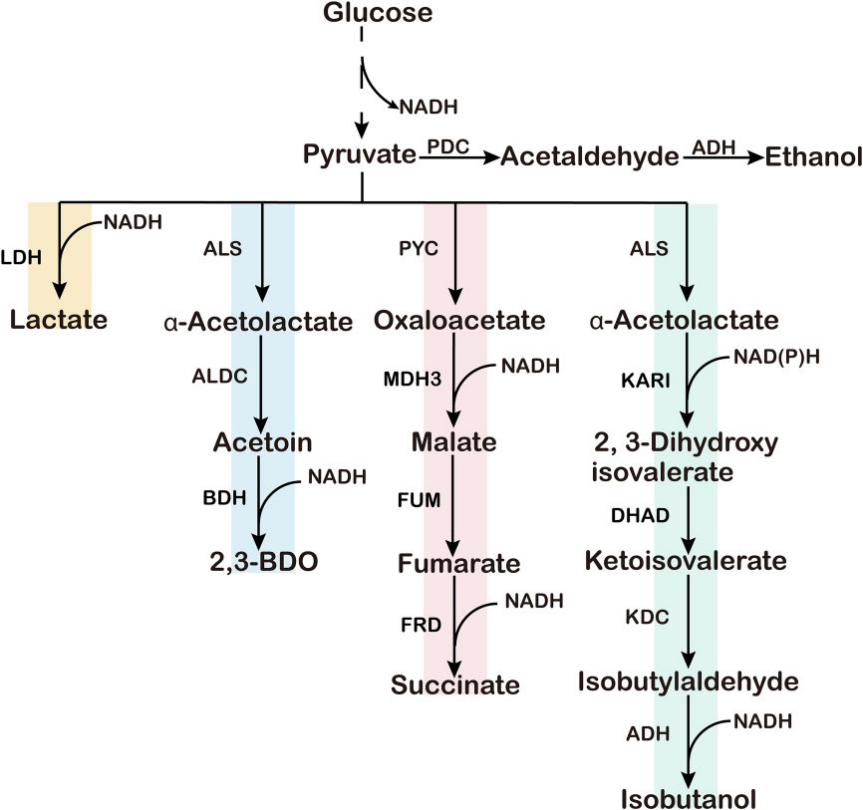
Nonethanol chemicals produced in Crabtree-negative *S. cerevisiae*. These chemicals include LA, 2,3-BDO, malic acid, succinic acid, and isobutanol.

PDC-negative yeast strains engineered by deleting PDC usually have excessive accumulation of pyruvate (Van Maris et al. 2004a, Wang et al. 2012), which is an important metabolic node connecting glycolysis, ethanol fermentation, and aerobic respiration. It is also a precursor of many important chemicals. Through metabolic engineering, pyruvate-producing strains can be engineered to produce other important compounds, which can also replace ethanol and consume NADH. Therefore, coupling product synthesis with NADH consumption can alleviate the redox imbalance caused by PDC deletion. Chemicals such as 2,3-BDO, LA, malic acid, and isobutanol have been produced (Fig. 5) (Table 1). LA, malic acid, and 2,3-BDO are suitable substitutes for ethanol production that consume the same amount of NADH. Succinic acid requires one more NADH via the reductive TCA (rTCA) pathway compared to malic acid. Isobutanol requires the participation of nicotinamide adenine dinucleotide phosphate (NADPH). In addition, enhanced carbon metabolism of Crabtree-negative strains also makes them potential cell factories to produce chemicals such as free fatty acids, 3-hydroxypropionic acid, and farnesene.

**Table 1.** Summary of strategies for nonethanol chemicals production in Crabtree-negative *S. cerevisiae*.

Compounds	Strains	Major metabolic engineering strategies	Titer (g l^−1^)	Yield (g g^−1^ carbon source)	Productivity (g l^−1^ h^−1^)	Conditions (carbon source, batch/fed-batch, vessel)	References
LA	AF297C	*∆PDC1, ∆ADH1*; express *LDH* from bovine	75	0.75	0.075	Glucose; batch; bioreactor	Tokuhiro et al. (2009)
	SP1130	*∆PDC1, ∆ADH1, ∆CYB2, ∆GPD1, ∆ALD6*; express *LDH* from *P. s. japonicas* and *B. tauru, mphF* and *eutE* from *E. coli*	142	0.89	3.55	Glucose; feed-batch; bioreactor	Song et al. (2016)
	YIP-J-C-D-A1	*∆PDC1, ∆PDC6, ∆ADH1, ∆JEN1, ∆CYB2, ∆DLD1*; express *Ldh* from *E.coli*	80	0.6	1.1	Glucose; feed-batch; bioreactor	Zhong et al. (2019)
	S.c-NO.2–100	*∆PDC1, ∆ADH1*; overexpres *LDH* from bovines, *eutE* from *E. coli, JEN1* from *S. cerevisiae*; evolution in media with lactate	121.5	0.81	1.69	Glucose; feed-batch; bioreactor	Zhu et al. (2022)
	TAM-L17	PH 2.4 tolerant starting strain; *∆PDC1, ∆PDC6, ∆ADH1, ∆CYB2, ∆GPD1, ∆GPD2, ∆JEN1, ∆NDE1, ∆NDE2*; overexpress *LDH* from *L. lactis, PfkA* from *E. coli, ADY2* from *S. cerevisiae*	192.3	0.78	1.6	Glucose; feed-batch; bioreactor	Liu et al. (2023)
2,3-BDO	YHI030	*∆PDC1, ∆PDC5, ∆PDC6, MTH1^L165F^*; overexpress *AlsLp* from *L. plantarum, aldcLlOp* from *L. lactis, BDH1* from from *S. cerevisiae*; evolution under semiaerobic conditions	81.0	0.27	0.161	Glucose; feed-batch; erlenmeyer flasks	Ishii et al. (2018)
	S5_mBDO4	*∆PDC1, ∆PDC5, ∆PDC6, ∆GPD1, ∆GPD2, ∆BDH1*; overexpress *budC* from *K. oxytoca, alsS* and *alsD* from *B. subtilis; NoxE* from *L. lactis; CtPDC1* from *C. tropicalis*	171.0	0.49	1.80	Glucose; feed-batch; bioreactor	Lee et al. (2022)
	BD5_G1CtPDC1_nox	*∆PDC1, ∆PDC5, ∆PDC6*; overexpress *alsS, alsD* from *B. subtilis, BDH1* from *S. cerevisiae, CtPDC1* from *C. tropicalis, NoxE* from *L. lactis*	154.3	0.404	1.98	Glucose; feed-batch; bioreactor	Kim et al. (2016)
	HGS37	*∆PDC1, ∆PDC5, ∆PDC6, ∆GPD2, ∆ORA1*; overexpress *AlsA, AlsD, bdhA* from *Bacillus subtilis, NoxE* from *L. lactis, CSTL1* from *C. albicans*; weakly express *GPD1*	130.64	0.47	1.58	Glucose; feed-batch; bioreactor	Huo et al. (2022)
	HGS50	*∆PDC1, ∆PDC5, ∆PDC6, ∆GPD2, ∆MPC1, ∆ORA1*; overexpress *AlsA, AlsD, bdhA* from *B.subtilis, NDE1* and *AOX1* from *H. capsulatum*; weakly express *GPD1*	121.04	0.48	1.57	Glucose; feed-batch; bioreactor	
Malic acid	RWB525	Glucose-tolerant, C2^−^ dependent PDC negative starter strain; overexpress *PYC2* and *MDH3* from *S. cerevisiae, SpMae1p* from *S. pombe*	59	0.31	0.19	Glucose; batch; flask	Zelle et al. (2008)
	CTMAE-pro	*∆PDC1, ∆ADH1, ∆GPD1, ∆GPD2*; overexpress *PYC1, PYC2, MDH3∆SKL* from *S. cerevisiae, SpMae1* from *S. pombe*	61.2	0.23	0.32	Xylose; feed-batch; bioreactor	Kang et al. (2022)
	W4209	Glucose-tolerant, C2^−^ dependent PDC negative starter strain; overexpress *PYC* from *A. flavus, MDH* from R. oryzae, Spmae* (K395R, K409R and K416R) from *S. pombe*	30.25	0.3	0.32	Glucose; batch; flask	Chen et al. (2017)
Succinic acid	TAM-6gpd1∆fum1∆	*∆PDC1, ∆PDC5, ∆PDC6, ∆GPD1, ∆FUM1*; overexpress *PYC2, MDH3* and *FRDS1* from *S. cerevisiae, pckA* from *M. succiniciproducens, fumC* from *E. coli, SpMae1* from *S. pombe*	2.2	0.044	0.0153	Glucose; batch; deep‐well plate	Zahoor et al. (2019)
	PMCFfg	*∆PDC1, ∆PDC5, ∆PDC6, ∆GPD1, ∆FUM1*; overexpress *PYC2, MDH3, FRDS1* from *S. cerevisiae, FumC* from *E. coli*	12.97	0.1297	0.108	Glucose; batch; bioreactor	Yan et al. (2014)
	SynENG010	Building synthetic energy systems through three modules: the pentose phosphate (PP) pathway cycle, the trans-hydrogenase cycle, the external respiratory chain; *∆PDC1, ∆PDC5, ∆PDC6, ∆PFK2*, downregulation of *PFK1*; overexpress *PYC2, MDH3∆SKL, FRD1* from *S. cerevisiae, FumC* from *E. coli, SpMae1* from *S. pombe*	3.3	——	——	Glucose;—;—	Yu et al. (2022)
Isobutanol	YEZ167-4	OptoEXP, OptoINVRT systems were constructed to realize light-induced gene expression and repression; *∆PDC1, ∆PDC5, ∆PDC6, ∆BAT1*; dynamic regulation of *PDC1* and *ILV2* expression	8.49	0.0535	0.0321	Glucose; feed-batch; bioreactor	Zhao et al. (2018)
	sJD107	*∆PDC1, ∆PDC5, ∆PDC6*; library design and screening of isobutanol pathway enzymes; switch the cofactor preference of the *LbIlvC* (*KARI*) from being NADPH-dependent to NADH-dependent	0.364	0.036	0.00505	Glucose; batch; culture tube	Gambacorta et al. (2022)

### LA

LA has a wide range of industrial applications and can be used to produce polylactic acid (PLA) (Yang et al. 2015). LA is a substitute for ethanol, and the same amount of NADH is required to synthesize LA. The conversion of pyruvate to lactate is achieved by expressing lactate dehydrogenase (Ldh) in strains in which ethanol synthesis is blocked. LA in the cytoplasm alters cellular composition, such as the sphingolipid composition in the plasma membrane, and affects cellular physiology and metabolism (Abbott et al. 2008). LA accumulates and is then exported through intracellular protons and acidic anions via the H^+^-ATPase, a process that consumes energy (Van Maris et al. 2004b). Therefore, it is crucial to increase LA efflux and enhance tolerance to LA, in addition to reducing byproduct accumulation.

Ethanol is the predominant byproduct. Its accumulation was reduced primarily by the deletion of *PDC* and *ADH* (Table 1). LDH has many sources, including *Plasmodium falciparum* (Novy et al. 2017), the bovines (Ishida et al. 2005), *Lactococcus lactis* (Liu et al. 2023), *L. mesenteroides* (Baek et al. 2016b), and others. Tokuhiro et al. (2009) replaced endogenous Pdc1 and Adh1 with bovine L-Ldh. The double mutant strain significantly increased LA production but the growth rate on glucose was reduced.

When *ADH1* was deleted, strain growth was impaired. This cannot be attributed solely to redox imbalance, but also to intracellular acetaldehyde accumulation. Acetaldehyde is not only toxic to cells, but also inhibits Ald activity through substrate inhibition (Eggert et al. 2012). This is also unfavorable in terms of intracellular acetyl-CoA supply. Song et al. (2016) introduced mphF and eutE from *E. coli* to construct an alternative acetyl-CoA synthesis pathway in a Pdc1, Adh1, Gpd1, Cyb2, and Ald6 deletion strain, and the lactate yield was elevated from 0.8 to 0.85 g g^−1^. The resulting strain was able to produce 142 g l^−1^ of LA.

The main factor limiting LA accumulation is its toxicity to yeast. When LA production is high, the intracellular accumulation of LA alters the cytoplasmic environment and transport engineering becomes particularly important (Pacheco et al. 2012). Jen1 mediates the uptake of lactate, acetate, and pyruvate (Casal et al. 2016). Zhong et al. (2019) deleted Jen1 to reduce cytoplasmic toxicity due to lactate accumulation in combination with the knockout of the lactate utilization genes l-lactate cytochrome-c oxidoreductase Cyb2, d-lactate dehydrogenase1 Dld1 in a strain with deletion of *PDC1, PDC6*, and *ADH1*. The resulting strain could produce 80 g l^−1^ of LA. Additionally, ALE is an effective strategy for screening highly acid-tolerant yeasts. Zhu et al. (2022) increased the LA level from 10 to 60 g l^−1^ by 12 consecutive passage cultivation cultures, and the evolved strain increased LA production by 17.5%.

NADH dehydrogenase Nde1 and Nde2 affect the availability of intracellular redox cofactors (Maeda et al. 2021). Liu et al. (2023) constructed a LA production pathway from an evolved strain that could tolerate pH 2.4. After the deletion of Nde1 and Nde2, cytoplasmic NADH was redistributed, and LA production increased from 50.5 to 63.3 g l^−1^. Combining the strategies of weakening the branching pathway, increasing the product output, and improving glucose utilization efficiency, the LA production in the 15 l bioreactor was increased to 192.3 g l^−1^ with a yield of 0.78 g g^−1^, which is the highest level reported so far in *S. cerevisiae* (Liu et al. 2023).

In summary, LA production has been significantly improved through metabolic engineering strategies such as reduction of ethanol accumulation, introduction of *LDH* genes, construction of alternative acetyl-CoA synthesis pathways, and enhancing the strain tolerance of LA. LA production has been improved significantly. To further decrease the cost of LA production, it is necessary to further improve the acid tolerance to enable LA production at low pH.

### 2,3-BDO

2,3-BDO is a chemical widely used in food, pharmaceuticals, cosmetics, and other industries (Zhang et al. 2017). It can be used to produce 1,3-butadiene to synthesize rubber (Lynch 2001, Syu 2001). The 2,3-BDO pathway involves three enzymes, acetolactate synthase (Als), acetolactate decarboxylase (Alsc), and 2,3-butanediol dehydrogenase (Bdh), and they catalyze the conversion of pyruvate into α-acetolactate, acetoin, and 2,3-BDO, respectively. The BDH-catalyzed reaction consumes one molecule of NADH. The production of 2,3-BDO in *S. cerevisiae* faces challenges related to the low productivity of the endogenous pathway, the accumulation of by-products and the imbalance of cofactor.

To achieve high yields of 2,3-BDO, the heterologous production pathway was introduced into *S. cerevisiae*. Kim et al. (2013b) introduced Als and Alsc from *Bacillus subtilis* and overexpressed endogenous 2,3-butanediol dehydrogenase (Bdh1), which combined with the deletion of *PDC1* and *PDC5* yielded 96.2 g l^−1^ 2,3-BDO. Ishii et al. (2018) screened a variety of sources of enzymes to enhance the conversion of pyruvate to 2,3-BDO, and ultimately identified highly active Als from *L. plantarum*, Aldc from *L. lactis*, combined with deletions of *PDC1, PDC5*, and *PDC6* to obtain 81.0 g l^−1^ of 2,3-BDO. In addition to heterologous expression of Als and Alsc, the introduction of heterologous Bdh was also introduced. Lee et al. (2022) disrupted endogenous Bdh1 in *S. cerevisiae* and introduced budC from *K. oxytoca* for meso-2,3-BDO production.

The main by-products of 2,3-BDO production in *S. cerevisiae* are also ethanol and glycerol. Researchers have explored various combinations of *PDC* and *ADH* deletions to prevent ethanol accumulation (Table 1). Glycerol was eliminated by the deletion of *GPD1* and *GPD2*. When all three *PDC* genes were knocked out, cytoplasmic acetyl-CoA was deficient. Adding 0.5 g l^−1^ ethanol to the culture medium improved the production of 2,3-BDO (Kim et al. 2015). Kim et al. (2016) introduced *PDC1* from *Candida tropicalis* (*CtPDC1*) into Crabtree-negative yeasts to minimize ethanol accumulation and ensure the availability of cytoplasmic acetyl-CoA. The optimized strain exhibited a 2.3-fold increase in productivity compared to the control and produced 121.8 g l^−1^ 2,3-BDO in fed-batch fermentation (Kim et al. 2016).

*Saccharomyces cerevisiae* maintains intracellular redox balance by producing ethanol and glycerol. When the ethanol pathway is blocked, glycerol production becomes the primary maintenance pathway. Glycolysis produces two molecules of NADH, while the 2,3-BDO production pathway consumes only one molecule of NADH. Deletion of *GPD1* and *GPD2* can reduce glycerol synthesis, but can also lead to an intracellular redox imbalance. To oxidize NADH, water-forming NADH oxidase can convert excess NADH to NAD^+^ to water. Kim and Hahn (2015) introduced *noxE* from *L. lactis*, which increased the 2,3-BDO productivity from 0.26 to 0.44 g l^−1^ h^−1^ compared to the control strain. Huo et al. (2022) obtained two high yielding strains by reducing glycerol production through two different approaches. One strain introduced NADH oxidase NoxE from *L. lactis*, knocked out *GPD2*, reduced the expression of *GPD1* and introduced sugar transporter-like gene (*STL1*) from *Candida albicans*, producing 130.64 g l^−1^ 2,3-BDO with a productivity 1.58 g l^−1^ h^−1^. Another strain expressed the homologous extramitochondrial NADH dehydrogenase Nde1 and the alternative oxidase Aox1 from *Histoplasma capsulatum*, combined with downregulation of *GPD1* and the deletion of *GPD2*, producing 121.04 g l^−1^ 2,3-BDO with a productivity of 1.57 g l^−1^ h^−1^.

Significant progress has been made in the production of 2,3-BDO by *S. cerevisiae* (Lee et al. 2021, Mitsui et al. 2022). Researchers have attempted to replace glucose with lower-cost biomass, such as xylose (Kim et al. 2017) and cassava hydrolysate (Lee and Seo 2019). However, the application of these substrates typically results in lower yields and productivity. Therefore, future work can focus on further optimization of the relevant pathways for efficient production of 2,3-BDO from these substrates.

### 1,4-dicarboxylic acids

Malic acid and succinic acid are dicarboxylic acids, both of which are listed in the US Department of Energy’s 2004 list of 12 high-value bio-based platform chemicals (Werpy et al. 2004). The rTCA pathway yields more malate and succinate than the oxidative TCA pathway and the glyoxalate cycle. In this pathway, pyruvate is carboxylated by pyruvate carboxylase (Pyc) to produce oxaloacetate, which is then reduced by malate dehydrogenase (Mdh) to produce malate. Malate is then converted sequentially by fumarase (Fum) and fumarate reductase (Frd) to produce succinate. In this pathway, the maximum theoretical yield of both malate and succinate is 2 mol mol^−1^ glucose.

In PDC-negative *S. cerevisiae*, Zelle et al. (2008) removed the signal peptide of endogenous malate dehydrogenase 3 (Mdh3) to localize it in the cytoplasm. The combination of overexpression of pyruvate carboxylase Pyc2 and the dicarboxylic acid transporter SpMae1 from *Schizosaccharomyces pombe* resulted in 59 g l^−1^ malate (Zelle et al. 2008). Chen et al. (2017) combined Pyc from *Aspergillus flavus*, Mdh from *Rhizopus oryzae*, and the dicarboxylic acid transporter protein SpMae1, elevating the titer to 11.86 g l^−1^. Three key mutations in SpMae1, K395R, K409R, and K416R to obtain deubiquitylated SpMae1* were identified, and the titer of the resulting strain was elevated to 22.14 g l^−1^. Subsequently, a strain that could produce 30.25 g l^−1^ malic acid was obtained by optimizing gene expression, but pyruvic acid accumulated to a high level, reaching 30.73 g l^−1^ (Chen et al. 2017). The above studies demonstrated that overexpression of carboxylase and transporter proteins was crucial for malate production in PDC-negative strains. However, pyruvate is still accumulated, and the flux from pyruvate to malate needs to be improved. In addition, Sun et al (2023) obtained a strain tolerant to pH 2.3 by ALE. Based on it, they constructed a strain that could produce 232.9 g l^−1^ malic acid, which is the highest titer reported to date. It highlights the importance of the evolution of acid tolerance for organic acid production.

Aside from malate, succinate-producing strains were also constructed. Yan et al. (2014) constructed succinate rTCA pathway in *S. cerevisiae*, which consists of Mdh3 that removes the last three amino acid residues to target to the cytoplasm, the fumarase fumC from *E. coli*, and the endogenous overexpression of the fumarate reductases Frds1 and pyruvate carboxylase Pyc2. Gpd1 is the main enzyme for glycerol production and Fum1 tends to catalyze the production of malic acid from fumaric acid, so both enzymes were knocked out. The strain produced 12.97 g l^−1^ succinate (Yan et al. 2014). Zahoor et al. (2019) also produced succinic acid via the rTCA pathway, and specifically introduced pckA, a Pep carboxykinase from *Mannheimia succiniciproducens*, an enzyme that converts PEP to OAA and fixes CO_2_. Yu et al. (2022) engineered a decarboxylation cycle in *S. cerevisiae* to provide reducing power, and the accumulation of succinate and glycerol demonstrated that the pathway produced NADH, with succinate reaching 3.3 g l^−1^. Although some studies have explored succinic acid production through rTCA pathway in *S. cerevisiae*, the current production of succinic acid still faces low yield and accumulation of by-products.

### Isobutanol

Isobutanol has a higher energy density than ethanol and can be used as a biofuel (Buijs et al. 2013, Roussos et al. 2019). The metabolic pathway of isobutanol involves five enzymes, including Als, ketoacid reductoisomerase (Kari), dihydroxyacid dehydratase (Dhad), α-ketoacid decarboxylase (Kdc), and alcohol dehydrogenase (Adh). Among them, the Kari is NADPH-dependent and the Adh is NADH-dependent. Isobutanol is a suitable substitute for ethanol because both require two reducing equivalents (Milne et al. 2016). Gambacorta et al. (2022) built a combinatorial pathway library of enzymes in isobutanol production, and determined the optimal source and expression level of the enzymes in the pathway with a titer of 364 mg l^−1^. However, the Kari-dependent cofactor is NADPH. Shifting the cofactor dependence of the enzyme from NADPH to NADH is important (Brinkmann-Chen et al. 2013). Therefore, Gambacorta et al. (2022) then worked on converting the cofactor preference of Kari from NADPH to NADH, but this approach failed to improve isobutanol production under aerobic conditions. Optogenetics combined with metabolic engineering were also performed to produce isobutanol (Zhao et al. 2018). Zhao et al. (2018) regulated Pdc1 and Als Ilv2 by the optogenetic circuits OptoEXP and OptoINVRT, respectively. In the presence of light, the *PDC1* gene was expressed and the cells grew. In dark condition, ethanol production was transformed into isobutanol accumulation. After optimizing the conditions, 8.49 ± 0.31 g l^−1^ of isobutanol was accumulated. This suggests that dynamic regulation is a promising strategy for balancing cell growth and product synthesis in isobutanol production. Integrating metabolic engineering approaches, optimizing enzyme efficiency and engineering cofactor utilization to obtain higher isobutanol production is expected.

### Other chemicals

Aside from pyruvate-derived chemicals, Crabtree-negative *S. cerevisiae* has also been employed to produce other compounds such as fatty acids and 3-hydroxypropionate. Yu et al. (2018) rewired the metabolic pathway for fatty acids production in a PDC-negative yeast. They first enhanced cytosolic acetyl-CoA and NADPH supply, and the resulting strain Y&Z036 produced 33.4 g l^−1^ FFA. The PDC-negative yeast was then created to reprogram yeast metabolism from alcoholic fermentation to lipogenesis. The PDC-negative strain produced 25 g l^−1^ FFA, demonstrating the potential of *S. cerevisiae* for efficient FFA production. Yao et al. (2023) found that Crabtree-negative *S. cerevisiae* had enhanced carbon metabolism and reduced protein translation. When using the strain to produce chemicals, the titers of 2,3-BDO, LA, *p*-coumaric acid, farnesene, lycopene, 3-hydroxypropionate, and fatty acids were significantly higher than in Crabtree-positive *S. cerevisiae*. These studies further highlight the versatility of Crabtree-negative *S. cerevisiae* as a microbial cell factory. Crabtree-negative yeasts generally have higher respiration and lower byproduct accumulation than Crabtree-positive yeasts, and they can provide more energy and precursors for biosynthesis.

## Summary and prospects

*Saccharomyces cerevisiae* with Crabtree effect can rapidly utilize glucose and convert it to ethanol, which is then slowly consumed as a carbon source. Although rapid utilization of carbon sources can result in rapid growth, ethanol production limits the carbon flux to the desired chemicals. Deletion of *PDC* blocks ethanol production, but disrupts NADH balance and abolishes cytoplasmic acetyl-CoA supply, which affects cell growth. ALE of *PDC*-deficient strains has identified several key mutations that can effectively mitigate the Crabtree effect by reducing glucose uptake or balancing glycolysis with respiratory metabolism. These mutations include Mth1^A81P^, Pyk1^R68*^, Med2^*432Y^, Oca5^S383*^, and others. Mutations such as Mth1^A81P^ and Pyk1^R68*^ reduce glucose uptake and glycolysis, while Med2^*432Y^ and Oca5^S383*^ refigure the metabolism from high glycolysis to elevated respiration. The metabolism refiguration contributes to restoring the intracellular NADH homeostasis and recover the growth.

Crabtree-negative yeasts avoid the carbon waste for ethanol production, thereby becoming potential cell factories for the biosynthesis of other chemicals. Through metabolic engineering, the production of chemicals such as 2,3-BDO, LA, and malic acid has reached to a relatively high level. Further engineering is required to improve the production of chemicals such as succinic acid and isobutanol. In addition, as acetyl-CoA supply can be optimized in Crabtree-negative yeasts, and these cell factories have great potential to produce acetyl-CoA derived chemicals such as fatty acids, farnesene and so on.

Although Crabtree-negative *S. cerevisiae* has been developed and employed for different chemicals production, these strains still exhibit several limitations. The growth has been restored to some extent by ALE, however, the recovery of growth is achieved at the expense of glucose uptake rate. It is still necessary to engineer the strain to improve the growth and glucose consumption rate. Alternatively, it is possible to create Crabtree-negative yeast through engineering yeast to use the carbon sources, such as xylose, glycerol, or even sucrose. As we know that high glucose concentration triggers glucose repression, which will in turn suppresses respiration-related genes. In contrast, when alternative carbon source is used, glucose repression will be eliminated and respiration can be activated.

In addition, dynamic regulation is an alternative way to eliminate ethanol production. Dynamic switches can respond to metabolic signals and provide timely feedback (Lalwani et al. 2018, Shen et al. 2019, Xiao et al. 2023). Optogenetics was applied to control *PDC1* expression based on light conditions. This could enable precise regulation of carbon fluxes, switching between ethanol-promoted growth and target product accumulation (Zhao et al. 2018). This balance between growth and product synthesis has been successfully demonstrated for isobutanol production. Similar approach holds great potential for dynamically controlling metabolic flux and maximizing production without sacrificing growth.

In summary, by eliminating the ethanol production pathway, the application potential of *S. cerevisiae* has been expanded beyond traditional fermentation products to include a diverse range of bio-based chemicals and high-value-added products. Meanwhile, the metabolic mechanism underlying the Crabtree effect has been gradually elucidated. We are confident that, through ongoing research efforts, Crabtree-negative *S. cerevisiae* will emerge as an efficient and flexible host for producing a broader spectrum of bio-based chemicals.

## References

[bib1] Abbott DA, Suir E, van Maris AJ et al. Physiological and transcriptional responses to high concentrations of lactic acid in anaerobic chemostat cultures of *Saccharomyces cerevisiae*. Appl Environ Microbiol. 2008;74:5759–68. 18676708 10.1128/AEM.01030-08 PMC2547041

[bib2] Aceituno FF, Orellana M, Torres J et al. Oxygen response of the wine yeast *Saccharomyces cerevisiae* EC1118 grown under carbon-sufficient, nitrogen-limited enological conditions. Appl Environ Microbiol. 2012;78:8340–52. 23001663 10.1128/AEM.02305-12 PMC3497381

[bib3] Agarwal PK, Uppada V, Noronha SB. Comparison of pyruvate decarboxylases from *Saccharomyces cerevisiae* and *Komagataella pastoris* (*Pichia pastoris*). Appl Microbiol Biotechnol. 2013;97:9439–49. 23423327 10.1007/s00253-013-4758-4

[bib4] Baek S-H, Kwon EY, Kim S-Y et al. GSF2 deletion increases lactic acid production by alleviating glucose repression in *Saccharomyces cerevisiae*. Sci Rep. 2016a;6:34812. 27708428 10.1038/srep34812 PMC5052599

[bib5] Baek SH, Kwon EY, Kim YH et al. Metabolic engineering and adaptive evolution for efficient production of D-lactic acid in *Saccharomyces cerevisiae*. Appl Microbiol Biotechnol. 2016b;100:2737–48. 26596574 10.1007/s00253-015-7174-0

[bib6] Bakker BM, Overkamp KM, van Maris AJ et al. Stoichiometry and compartmentation of NADH metabolism in *Saccharomyces cerevisiae*. FEMS Microbiol Rev. 2001;25:15–37. 11152939 10.1111/j.1574-6976.2001.tb00570.x

[bib7] Boer VM, de Winde JH, Pronk JT et al. The genome-wide transcriptional responses of *Saccharomyces cerevisiae* grown on glucose in aerobic chemostat cultures limited for carbon, nitrogen, phosphorus, or sulfur. J Biol Chem. 2003;278:3265–74. 12414795 10.1074/jbc.M209759200

[bib8] Bogorad IW, Lin TS, Liao JC. Synthetic non-oxidative glycolysis enables complete carbon conservation. Nature. 2013;502:693–7. 24077099 10.1038/nature12575

[bib9] Brinkmann-Chen S, Flock T, Cahn JK et al. General approach to reversing ketol-acid reductoisomerase cofactor dependence from NADPH to NADH. Proc Natl Acad Sci USA. 2013;110:10946–51. 23776225 10.1073/pnas.1306073110 PMC3704004

[bib10] Buijs NA, Siewers V, Nielsen J. Advanced biofuel production by the yeast *Saccharomyces cerevisiae*. Curr Opin Chem Biol. 2013;17:480–8. 23628723 10.1016/j.cbpa.2013.03.036

[bib11] Casal M, Queirós O, Talaia G et al. Carboxylic acids plasma membrane transporters in *Saccharomyces cerevisiae*. Adv Exp Med Biol. 2016;892:229–51. 26721276 10.1007/978-3-319-25304-6_9

[bib12] Chen H, Blum JE, Thalacker-Mercer A et al. Impact of the whole genome duplication event on PYK activity and effects of a PYK1 mutation on metabolism in *S. cerevisiae*. Front Mol Biosci. 2021;8:656461. 33796550 10.3389/fmolb.2021.656461 PMC8007964

[bib13] Chen X, Wang Y, Dong X et al. Engineering rTCA pathway and C4-dicarboxylate transporter for L-malic acid production. Appl Microbiol Biotechnol. 2017;101:4041–52. 28229207 10.1007/s00253-017-8141-8

[bib14] Chen Y, Nielsen J. Energy metabolism controls phenotypes by protein efficiency and allocation. Proc Natl Acad Sci USA. 2019;116:17592–7. 31405984 10.1073/pnas.1906569116 PMC6717264

[bib15] Chen Y, Nielsen J. Yeast has evolved to minimize protein resource cost for synthesizing amino acids. Proc Natl Acad Sci USA. 2022;119:e2114622119. 35042799 10.1073/pnas.2114622119 PMC8795554

[bib16] Chen Y, Zhang Y, Siewers V et al. Ach1 is involved in shuttling mitochondrial acetyl units for cytosolic C2 provision in *Saccharomyces cerevisiae* lacking pyruvate decarboxylase. FEMS Yeast Res. 2015;15:fov015. 25852051 10.1093/femsyr/fov015

[bib17] Conrad M, Schothorst J, Kankipati HN et al. Nutrient sensing and signaling in the yeast *Saccharomyces cerevisiae*. FEMS Microbiol Rev. 2014;38:254–99. 24483210 10.1111/1574-6976.12065 PMC4238866

[bib18] Cuello RA, Flores Montero KJ, Mercado LA et al. Construction of low-ethanol-wine yeasts through partial deletion of the *Saccharomyces cerevisiae* PDC2 gene. AMB Expr. 2017;7:67. 10.1186/s13568-017-0369-2 PMC5360750 28324615

[bib19] Dai Z, Huang M, Chen Y et al. Global rewiring of cellular metabolism renders *Saccharomyces cerevisiae* Crabtree negative. Nat Commun. 2018;9:3059. 30076310 10.1038/s41467-018-05409-9 PMC6076296

[bib20] De Smidt O, Du Preez JC, Albertyn J. The alcohol dehydrogenases of *Saccharomyces cerevisiae*: a comprehensive review. FEMS Yeast Res. 2008;8:967–78. 18479436 10.1111/j.1567-1364.2008.00387.x

[bib21] De Smidt O, Du Preez JC, Albertyn J. Molecular and physiological aspects of alcohol dehydrogenases in the ethanol metabolism of *Saccharomyces cerevisiae*. FEMS Yeast Res. 2012;12:33–47. 22094012 10.1111/j.1567-1364.2011.00760.x

[bib22] Díaz-Ruiz R, Avéret N, Araiza D et al. Mitochondrial oxidative phosphorylation is regulated by fructose 1,6-bisphosphate. A possible role in Crabtree effect induction?. J Biol Chem. 2008;283:26948–55. 18682403 10.1074/jbc.M800408200

[bib23] Dickinson JR, Salgado LEJ, Hewlins MJ. The catabolism of amino acids to long chain and complex alcohols in *Saccharomyces cerevisiae*. J Biol Chem. 2003;278:8028–34. 12499363 10.1074/jbc.M211914200

[bib24] Dotson MR, Yuan CX, Roeder RG et al. Structural organization of yeast and mammalian mediator complexes. Proc Natl Acad Sci USA. 2000;97:14307–10. 11114191 10.1073/pnas.260489497 PMC18914

[bib25] Eberhardt I, Cederberg H, Li H et al. Autoregulation of yeast pyruvate decarboxylase gene expression requires the enzyme but not its catalytic activity. Eur J Biochem. 1999;262:191–201. 10231381 10.1046/j.1432-1327.1999.00370.x

[bib26] Eggert MW, Byrne ME, Chambers RP. Kinetic involvement of acetaldehyde substrate inhibition on the rate equation of yeast aldehyde dehydrogenase. Appl Biochem Biotechnol. 2012;168:824–33. 22915233 10.1007/s12010-012-9822-5

[bib27] Flikweert MT, de Swaaf M, van Dijken JP et al. Growth requirements of pyruvate-decarboxylase-negative *Saccharomyces cerevisiae*. FEMS Microbiol Lett. 1999;174:73–79. 10234824 10.1111/j.1574-6968.1999.tb13551.x

[bib28] Flikweert MT, Van Der Zanden L, Janssen WM et al. Pyruvate decarboxylase: an indispensable enzyme for growth of *Saccharomyces cerevisiae* on glucose. Yeast. 1996;12:247–57. 8904337 10.1002/(SICI)1097-0061(19960315)12:3%3C247::AID-YEA911%3E3.0.CO;2-I

[bib29] Franken J, Kroppenstedt S, Swiegers JH et al. Carnitine and carnitine acetyltransferases in the yeast *Saccharomyces cerevisiae*: a role for carnitine in stress protection. Curr Genet. 2008;53:347–60. 18427809 10.1007/s00294-008-0191-0

[bib30] Gambacorta FV, Dietrich JJ, Baerwald JJ et al. Combinatorial library design for improving isobutanol production in *Saccharomyces cerevisiae*. Front Bioeng Biotechnol. 2022;10:1080024. 36532572 10.3389/fbioe.2022.1080024 PMC9755324

[bib31] Gancedo JM . The early steps of glucose signalling in yeast. FEMS Microbiol Rev. 2008;32:673–704. 18559076 10.1111/j.1574-6976.2008.00117.x

[bib32] Grüning N-M, Rinnerthaler M, Bluemlein K et al. Pyruvate kinase triggers a metabolic feedback loop that controls redox metabolism in respiring cells. Cell Metab. 2011;14:415–27. 21907146 10.1016/j.cmet.2011.06.017 PMC3202625

[bib33] Guirimand G, Kulagina N, Papon N et al. Innovative tools and strategies for optimizing yeast cell factories. Trends Biotechnol. 2021;39:488–504. 33008642 10.1016/j.tibtech.2020.08.010

[bib34] Hagman A, Piškur J. A study on the fundamental mechanism and the evolutionary driving forces behind aerobic fermentation in yeast. PLoS One. 2015;10:e0116942. 25617754 10.1371/journal.pone.0116942 PMC4305316

[bib35] Hou J, Qiu C, Shen Y et al. Engineering of *Saccharomyces cerevisiae* for the efficient co-utilization of glucose and xylose. FEMS Yeast Res. 2017;17:100084. 10.1093/femsyr/fox034 28582494

[bib36] Huo G, Foulquié-Moreno MR, Thevelein JM. Development of an industrial yeast strain for efficient production of 2,3-butanediol. Microb Cell Fact. 2022;21:199. 36175998 10.1186/s12934-022-01924-z PMC9520875

[bib37] Ida Y, Furusawa C, Hirasawa T et al. Stable disruption of ethanol production by deletion of the genes encoding alcohol dehydrogenase isozymes in *Saccharomyces cerevisiae*. J Biosci Bioeng. 2012;113:192–5. 22033067 10.1016/j.jbiosc.2011.09.019

[bib38] Iosue CL, Ugras JM, Bajgain Y et al. Pyruvate decarboxylase and thiamine biosynthetic genes are regulated differently by Pdc2 in *S. cerevisiae* and *C. glabrata*. PLoS One. 2023;18:e0286744. 37285346 10.1371/journal.pone.0286744 PMC10246790

[bib39] Ishida N, Saitoh S, Tokuhiro K et al. Efficient production of L-lactic acid by metabolically engineered *Saccharomyces cerevisiae* with a genome-integrated L-lactate dehydrogenase gene. Appl Environ Microbiol. 2005;71:1964–70. 15812027 10.1128/AEM.71.4.1964-1970.2005 PMC1082537

[bib40] Ishii J, Morita K, Ida K et al. A pyruvate carbon flux tugging strategy for increasing 2,3-butanediol production and reducing ethanol subgeneration in the yeast *Saccharomyces cerevisiae*. Biotechnol Biofuels. 2018;11:180. 29983743 10.1186/s13068-018-1176-y PMC6020211

[bib41] Jacobi T, Kratzer DA, Plapp BV. Substitution of both histidines in the active site of yeast alcohol dehydrogenase 1 exposes underlying pH dependencies. Chem Biol Interact. 2024;394:110992. 38579923 10.1016/j.cbi.2024.110992 PMC11090211

[bib42] Jouandot D, Roy A, Kim JH. Functional dissection of the glucose signaling pathways that regulate the yeast glucose transporter gene (HXT) repressor Rgt1. J Cell Biochem. 2011;112:3268–75. 21748783 10.1002/jcb.23253 PMC3341738

[bib43] Kanehisa M, Goto S, Sato Y et al. Data, information, knowledge and principle: back to metabolism in KEGG. Nucl Acids Res. 2014;42:D199–205. 24214961 10.1093/nar/gkt1076 PMC3965122

[bib44] Kang NK, Lee JW, Ort DR et al. L-malic acid production from xylose by engineered *Saccharomyces cerevisiae*. Biotechnol J. 2022;17:e2000431. 34390209 10.1002/biot.202000431

[bib45] Kayikci Ö, Nielsen J. Glucose repression in *Saccharomyces cerevisiae*. FEMS Yeast Res. 2015;15:fov068. 26205245 10.1093/femsyr/fov068 PMC4629793

[bib46] Kim J-H, Bloor D, Rodriguez R et al. Casein kinases are required for the stability of the glucose-sensing receptor Rgt2 in yeast. Sci Rep. 2022;12:1598. 35102180 10.1038/s41598-022-05569-1 PMC8803954

[bib47] Kim J-H, Rodriguez R. Glucose regulation of the paralogous glucose sensing receptors Rgt2 and Snf3 of the yeast *Saccharomyces cerevisiae*. Biochim Biophys Acta Gen Sub. 2021;1865:129881. 10.1016/j.bbagen.2021.129881 33617932

[bib48] Kim JW, Kim J, Seo SO et al. Enhanced production of 2,3-butanediol by engineered *Saccharomyces cerevisiae* through fine-tuning of pyruvate decarboxylase and NADH oxidase activities. Biotechnol Biofuels. 2016;9:265. 27990176 10.1186/s13068-016-0677-9 PMC5148919

[bib49] Kim JW, Seo SO, Zhang GC et al. Expression of *Lactococcus lactis* NADH oxidase increases 2,3-butanediol production in Pdc-deficient *Saccharomyces cerevisiae*. Bioresour Technol. 2015;191:512–9. 25769689 10.1016/j.biortech.2015.02.077

[bib50] Kim S, Hahn JS. Efficient production of 2,3-butanediol in *Saccharomyces cerevisiae* by eliminating ethanol and glycerol production and redox rebalancing. Metab Eng. 2015;31:94–101. 26226562 10.1016/j.ymben.2015.07.006

[bib51] Kim SJ, Seo SO, Jin YS et al. Production of 2,3-butanediol by engineered *Saccharomyces cerevisiae*. Bioresour Technol. 2013;146:274–81. 23941711 10.1016/j.biortech.2013.07.081

[bib52] Kim S-J, Seo S-O, Jin Y-S et al. Production of 2, 3-butanediol by engineered *Saccharomyces cerevisiae*. Bioresour Technol. 2013;146:274–81. 23941711 10.1016/j.biortech.2013.07.081

[bib53] Kim SJ, Sim HJ, Kim JW et al. Enhanced production of 2,3-butanediol from xylose by combinatorial engineering of xylose metabolic pathway and cofactor regeneration in pyruvate decarboxylase-deficient *Saccharomyces cerevisiae*. Bioresour Technol. 2017;245:1551–7. 28651874 10.1016/j.biortech.2017.06.034

[bib54] Kozak BU, van Rossum HM, Benjamin KR et al. Replacement of the *Saccharomyces cerevisiae* acetyl-CoA synthetases by alternative pathways for cytosolic acetyl-CoA synthesis. Metab Eng. 2014;21:46–59. 24269999 10.1016/j.ymben.2013.11.005

[bib55] Kozak BU, van Rossum HM, Luttik MA et al. Engineering acetyl coenzyme A supply: functional expression of a bacterial pyruvate dehydrogenase complex in the cytosol of *Saccharomyces cerevisiae*. mBio. 2014;5:e01696–14. 25336454 10.1128/mBio.01696-14 PMC4212835

[bib56] Krivoruchko A, Zhang Y, Siewers V et al. Microbial acetyl-CoA metabolism and metabolic engineering. Metab Eng. 2015;28:28–42. 25485951 10.1016/j.ymben.2014.11.009

[bib57] Kusano M, Sakai Y, Kato N et al. Hemiacetal dehydrogenation activity of alcohol dehydrogenases in *Saccharomyces cerevisiae*. Biosci Biotechnol Biochem. 1998;62:1956–61. 9836432 10.1271/bbb.62.1956

[bib58] Lafuente MJ, Gancedo C, Jauniaux JC et al. Mth1 receives the signal given by the glucose sensors Snf3 and Rgt2 in *Saccharomyces cerevisiae*. Mol Microbiol. 2000;35:161–72. 10632886 10.1046/j.1365-2958.2000.01688.x

[bib59] Lalwani MA, Zhao EM, Avalos JL. Current and future modalities of dynamic control in metabolic engineering. Curr Opin Biotechnol. 2018;52:56–65. 29574344 10.1016/j.copbio.2018.02.007

[bib60] Lee JW, Lee YG, Jin YS et al. Metabolic engineering of non-pathogenic microorganisms for 2,3-butanediol production. Appl Microbiol Biotechnol. 2021;105:5751–67. 34287658 10.1007/s00253-021-11436-2

[bib61] Lee YG, Bae JM, Kim SJ. Enantiopure meso-2,3-butanediol production by metabolically engineered *Saccharomyces cerevisiae* expressing 2,3-butanediol dehydrogenase from *Klebsiella oxytoca*. J Biotechnol. 2022;354:1–9. 35644291 10.1016/j.jbiotec.2022.05.001

[bib62] Lee YG, Seo JH. Production of 2,3-butanediol from glucose and cassava hydrolysates by metabolically engineered industrial polyploid *Saccharomyces cerevisiae*. Biotechnol Biofuels. 2019;12:204. 31485270 10.1186/s13068-019-1545-1 PMC6714309

[bib63] Lian J, Chao R, Zhao H. Metabolic engineering of a *Saccharomyces cerevisiae* strain capable of simultaneously utilizing glucose and galactose to produce enantiopure (2R,3R)-butanediol. Metab Eng. 2014;23:92–99. 24525332 10.1016/j.ymben.2014.02.003

[bib64] Lian J, Si T, Nair NU et al. Design and construction of acetyl-CoA overproducing *Saccharomyces cerevisiae* strains. Metab Eng. 2014;24:139–49. 24853351 10.1016/j.ymben.2014.05.010

[bib65] Liu T, Sun L, Zhang C et al. Combinatorial metabolic engineering and process optimization enables highly efficient production of L-lactic acid by acid-tolerant *Saccharomyces cerevisiae*. Bioresour Technol. 2023;379:129023. 37028528 10.1016/j.biortech.2023.129023

[bib66] Lynch J . BD monomer and elastomer production processes. Chem Biol Interact. 2001;135-136:147–53. 11397387 10.1016/s0009-2797(01)00187-9

[bib67] Maeda T, Koch-Koerfges A, Bott M. Relevance of NADH dehydrogenase and alternative two-enzyme systems for growth of *Corynebacterium glutamicum* with glucose, lactate, and acetate. Front Bioeng Biotechnol. 2021;8:621213. 33585420 10.3389/fbioe.2020.621213 PMC7874156

[bib68] Malina C, Yu R, Björkeroth J et al. Adaptations in metabolism and protein translation give rise to the Crabtree effect in yeast. Proc Natl Acad Sci USA. 2021;118:e2112836118. 34903663 10.1073/pnas.2112836118 PMC8713813

[bib69] Martinez-Ortiz C, Carrillo-Garmendia A, Correa-Romero BF et al. SNF1 controls the glycolytic flux and mitochondrial respiration. Yeast. 2019;36:487–94. 31074533 10.1002/yea.3399

[bib70] Mathiasen DP, Lisby M. Cell cycle regulation of homologous recombination in *Saccharomyces cerevisiae*. FEMS Microbiol Rev. 2014;38:172–84. 24483249 10.1111/1574-6976.12066

[bib71] Mathy G, Navet R, Gerkens P et al. *Saccharomyces cerevisiae* mitoproteome plasticity in response to recombinant alternative ubiquinol oxidase. J Proteome Res. 2006;5:339–48. 16457600 10.1021/pr050346e

[bib72] Metzl-Raz E, Kafri M, Yaakov G et al. Principles of cellular resource allocation revealed by condition-dependent proteome profiling. eLife. 2017;6:e28034. 28857745 10.7554/eLife.28034 PMC5578734

[bib73] Milne N, Wahl SA, van Maris AJA et al. Excessive by-product formation: a key contributor to low isobutanol yields of engineered *Saccharomyces cerevisiae* strains. Metab Eng Commun. 2016;3:39–51. 29142820 10.1016/j.meteno.2016.01.002 PMC5678825

[bib74] Mitsui R, Yamada R, Matsumoto T et al. Bioengineering for the industrial production of 2,3-butanediol by the yeast, *Saccharomyces cerevisiae*. World J Microbiol Biotechnol. 2022;38:38. 35018511 10.1007/s11274-021-03224-x

[bib75] Mojzita D, Hohmann S. Pdc2 coordinates expression of the THI regulon in the yeast *Saccharomyces cerevisiae*. Mol Genet Genomics. 2006;276:147–61. 16850348 10.1007/s00438-006-0130-z

[bib76] Monschau N, Stahmann KP, Sahm H et al. Identification of *Saccharomyces cerevisiae* GLY1 as a threonine aldolase: a key enzyme in glycine biosynthesis. FEMS Microbiol Lett. 1997;150:55–60. 9163906 10.1111/j.1574-6968.1997.tb10349.x

[bib77] Moriya H, Johnston M. Glucose sensing and signaling in *Saccharomyces cerevisiae* through the Rgt2 glucose sensor and casein kinase I. Proc Natl Acad Sci USA. 2004;101:1572–7. 14755054 10.1073/pnas.0305901101 PMC341776

[bib78] Nett R . Vaccine-enhancing plant extract could be mass produced in yeast. Nature. 2024;629:760–1. 38719958 10.1038/d41586-024-01210-5

[bib79] Nevoigt E . Progress in metabolic engineering of *Saccharomyces cerevisiae*. Microbiol Mol Biol Rev. 2008;72:379–412. 18772282 10.1128/MMBR.00025-07 PMC2546860

[bib80] Nielsen J, Larsson C, van Maris A et al. Metabolic engineering of yeast for production of fuels and chemicals. Curr Opin Biotechnol. 2013;24:398–404. 23611565 10.1016/j.copbio.2013.03.023

[bib81] Nielsen J . Yeast systems biology: model organism and cell factory. Biotechnol J. 2019;14:e1800421. 30925027 10.1002/biot.201800421

[bib82] Nilsson A, Nielsen J. Metabolic trade-offs in yeast are caused by F1F0-ATP synthase. Sci Rep. 2016;6:22264. 26928598 10.1038/srep22264 PMC4772093

[bib83] Nosaka K, Esaki H, Onozuka M et al. Facilitated recruitment of Pdc2p, a yeast transcriptional activator, in response to thiamin starvation. FEMS Microbiol Lett. 2012;330:140–7. 22404710 10.1111/j.1574-6968.2012.02543.x

[bib84] Novy V, Brunner B, Müller G et al. Toward “homolactic” fermentation of glucose and xylose by engineered *Saccharomyces cerevisiae* harboring a kinetically efficient l-lactate dehydrogenase within pdc1-pdc5 deletion background. Biotech Bioeng. 2017;114:163–71. 10.1002/bit.26048 27426989

[bib85] Oud B, Flores C-L, Gancedo C et al. An internal deletion in MTH1 enables growth on glucose of pyruvate-decarboxylase negative, non-fermentative *Saccharomyces cerevisiae*. Microb Cell Fact. 2012;11:1–10. 22978798 10.1186/1475-2859-11-131 PMC3503853

[bib86] Pacheco A, Talaia G, Sá-Pessoa J et al. Lactic acid production in *Saccharomyces cerevisiae* is modulated by expression of the monocarboxylate transporters Jen1 and Ady2. FEMS Yeast Res. 2012;12:375–81. 22260735 10.1111/j.1567-1364.2012.00790.x

[bib87] Pangestu R, Kahar P, Kholida LN et al. Harnessing originally robust yeast for rapid lactic acid bioproduction without detoxification and neutralization. Sci Rep. 2022;12:13645. 35953496 10.1038/s41598-022-17737-4 PMC9372150

[bib88] Persson S, Shashkova S, Österberg L et al. Modelling of glucose repression signalling in yeast *Saccharomyces cerevisiae*. FEMS Yeast Res. 2022;22:foac012. 35238938 10.1093/femsyr/foac012 PMC8916112

[bib89] Pfeiffer T, Morley A. An evolutionary perspective on the Crabtree effect. Front Mol Biosci. 2014;1:17. 25988158 10.3389/fmolb.2014.00017 PMC4429655

[bib90] Pham T, Walden E, Huard S et al. Fine-tuning acetyl-CoA carboxylase 1 activity through localization: functional genomics reveals a role for the lysine acetyltransferase NuA4 and sphingolipid metabolism in regulating Acc1 activity and localization. Genetics. 2022;221:iyac086. 35608294 10.1093/genetics/iyac086 PMC9339284

[bib91] Piskur J, Rozpedowska E, Polakova S et al. How did Saccharomyces evolve to become a good brewer?. Trends Genet. 2006;22:183–6. 16499989 10.1016/j.tig.2006.02.002

[bib92] Polish JA, Kim J-H, Johnston M. How the Rgt1 transcription factor of *Saccharomyces cerevisiae* is regulated by glucose. Genetics. 2005;169:583–94. 15489524 10.1534/genetics.104.034512 PMC1449106

[bib93] Pretorius IS . Synthetic genome engineering forging new frontiers for wine yeast. Crit Rev Biotechnol. 2017;37:112–36. 27535766 10.1080/07388551.2016.1214945

[bib94] Pronk JT, Yde Steensma H, Van Dijken JP. Pyruvate metabolism in *Saccharomyces cerevisiae*. Yeast. 1996;12:1607–33. 9123965 10.1002/(sici)1097-0061(199612)12:16<1607::aid-yea70>3.0.co;2-4

[bib95] Qi X, Wang Z, Lin Y et al. Elucidation and engineering mitochondrial respiratory-related genes for improving bioethanol production at high temperature in *Saccharomyces cerevisiae*. Eng Microbiol. 2024;4:100108. 39629328 10.1016/j.engmic.2023.100108 PMC11610969

[bib96] Qin N, Li L, Ji X et al. Rewiring central carbon metabolism ensures increased provision of acetyl-CoA and NADPH required for 3-OH-propionic acid production. ACS Synth Biol. 2020;9:3236–44. 33186034 10.1021/acssynbio.0c00264

[bib97] Qin N, Li L, Ji X et al. Flux regulation through glycolysis and respiration is balanced by inositol pyrophosphates in yeast. Cell. 2023;186:748–763.e15. 36758548 10.1016/j.cell.2023.01.014

[bib98] Qiu Y, Wu M, Bao H et al. Engineering of *Saccharomyces cerevisiae* for co-fermentation of glucose and xylose: current state and perspectives. Eng Microbiol. 2023;3:100084. 39628931 10.1016/j.engmic.2023.100084 PMC11611035

[bib99] Rodriguez S, Denby CM, Van Vu T et al. ATP citrate lyase mediated cytosolic acetyl-CoA biosynthesis increases mevalonate production in *Saccharomyces cerevisiae*. Microb Cell Fact. 2016;15:48. 26939608 10.1186/s12934-016-0447-1 PMC4778282

[bib100] Rosas Lemus M, Roussarie E, Hammad N et al. The role of glycolysis-derived hexose phosphates in the induction of the Crabtree effect. J Biol Chem. 2018;293:12843–54. 29907566 10.1074/jbc.RA118.003672 PMC6102143

[bib101] Roussos A, Misailidis N, Koulouris A et al. A feasibility study of cellulosic isobutanol production—process simulation and economic analysis. Processes. 2019;7:667.

[bib102] Roy A, Hashmi S, Li Z et al. The glucose metabolite methylglyoxal inhibits expression of the glucose transporter genes by inactivating the cell surface glucose sensors Rgt2 and Snf3 in yeast. MBoC. 2016;27:862–71. 26764094 10.1091/mbc.E15-11-0789 PMC4803311

[bib103] Roy A, Shin YJ, Cho KH et al. Mth1 regulates the interaction between the Rgt1 repressor and the Ssn6-Tup1 corepressor complex by modulating PKA-dependent phosphorylation of Rgt1. MBoC. 2013;24:1493–503. 23468525 10.1091/mbc.E13-01-0047 PMC3639059

[bib104] Rozpędowska E, Hellborg L, Ishchuk OP et al. Parallel evolution of the make-accumulate-consume strategy in *Saccharomyces* and *Dekkera* yeasts. Nat Commun. 2011;2:302. 21556056 10.1038/ncomms1305 PMC3112538

[bib105] Seeboth PG, Bohnsack K, Hollenberg CP. pdc1(0) mutants of *Saccharomyces cerevisiae* give evidence for an additional structural PDC gene: cloning of PDC5, a gene homologous to PDC1. J Bacteriol. 1990;172:678–85. 2404950 10.1128/jb.172.2.678-685.1990 PMC208492

[bib106] Sharma J, Kumar V, Prasad R et al. Engineering of *Saccharomyces cerevisiae* as a consolidated bioprocessing host to produce cellulosic ethanol: recent advancements and current challenges. Biotechnol Adv. 2022;56:107925. 35151789 10.1016/j.biotechadv.2022.107925

[bib107] Shen X, Wang J, Li C et al. Dynamic gene expression engineering as a tool in pathway engineering. Curr Opin Biotechnol. 2019;59:122–9. 31063878 10.1016/j.copbio.2019.03.019

[bib108] Shen Y, Dinh HV, Cruz ER et al. Mitochondrial ATP generation is more proteome efficient than glycolysis. Nat Chem Biol. 2024;20:1123–32. 10.1038/s41589-024-01571-y. 38448734 PMC11925356

[bib109] Snowdon C, Johnston M. A novel role for yeast casein kinases in glucose sensing and signaling. MBoC. 2016;27:3369–75. 27630263 10.1091/mbc.E16-05-0342 PMC5170868

[bib110] Song JY, Park JS, Kang CD et al. Introduction of a bacterial acetyl-CoA synthesis pathway improves lactic acid production in *Saccharomyces cerevisiae*. Metab Eng. 2016;35:38–45. 26384570 10.1016/j.ymben.2015.09.006

[bib111] Sun L, Zhang Q, Kong X et al. Highly efficient neutralizer-free L-malic acid production using engineered *Saccharomyces cerevisiae*. Bioresour Technol. 2023;370:128580. 36608859 10.1016/j.biortech.2023.128580

[bib112] Syu MJ . Biological production of 2,3-butanediol. Appl Microbiol Biotechnol. 2001;55:10–18. 11234948 10.1007/s002530000486

[bib113] Tokuhiro K, Ishida N, Nagamori E et al. Double mutation of the PDC1 and ADH1 genes improves lactate production in the yeast *Saccharomyces cerevisiae* expressing the bovine lactate dehydrogenase gene. Appl Microbiol Biotechnol. 2009;82:883–90. 19122995 10.1007/s00253-008-1831-5

[bib114] Van de Peppel J, Kettelarij N, van Bakel H et al. Mediator expression profiling epistasis reveals a signal transduction pathway with antagonistic submodules and highly specific downstream targets. Mol Cell. 2005;19:511–22. 16109375 10.1016/j.molcel.2005.06.033

[bib115] Van Maris AJ, Geertman JM, Vermeulen A et al. Directed evolution of pyruvate decarboxylase-negative *Saccharomyces cerevisiae*, yielding a C2-independent, glucose-tolerant, and pyruvate-hyperproducing yeast. Appl Environ Microbiol. 2004;70:159–66. 14711638 10.1128/AEM.70.1.159-166.2004 PMC321313

[bib116] Van Maris AJ, Luttik MA, Winkler AA et al. Overproduction of threonine aldolase circumvents the biosynthetic role of pyruvate decarboxylase in glucose-limited chemostat cultures of *Saccharomyces cerevisiae*. Appl Environ Microbiol. 2003;69:2094–9. 12676688 10.1128/AEM.69.4.2094-2099.2003 PMC154831

[bib117] Van Maris AJ, Winkler AA, Porro D et al. Homofermentative lactate production cannot sustain anaerobic growth of engineered *Saccharomyces cerevisiae*: possible consequence of energy-dependent lactate export. Appl Environ Microbiol. 2004;70:2898–905. 15128549 10.1128/AEM.70.5.2898-2905.2004 PMC404449

[bib118] Van Roermund CW, Hettema EH, van den Berg M et al. Molecular characterization of carnitine-dependent transport of acetyl-CoA from peroxisomes to mitochondria in *Saccharomyces cerevisiae* and identification of a plasma membrane carnitine transporter, Agp2p. Embo J. 1999;18:5843–52. 10545096 10.1093/emboj/18.21.5843 PMC1171650

[bib119] Van Rossum HM, Kozak BU, Niemeijer MS et al. Requirements for carnitine shuttle-mediated translocation of mitochondrial acetyl moieties to the yeast cytosol. mBio. 2016;7:e00520–16. 27143389 10.1128/mBio.00520-16 PMC4959659

[bib120] Vemuri GN, Eiteman MA, McEwen JE et al. Increasing NADH oxidation reduces overflow metabolism in *Saccharomyces cerevisiae*. Proc Natl Acad Sci USA. 2007;104:2402–7. 17287356 10.1073/pnas.0607469104 PMC1892921

[bib121] Verschueren KHG, Blanchet C, Felix J et al. Structure of ATP citrate lyase and the origin of citrate synthase in the Krebs cycle. Nature. 2019;568:571–5. 30944476 10.1038/s41586-019-1095-5

[bib122] Wang S, Zhao F, Yang M et al. Metabolic engineering of *Saccharomyces cerevisiae* for the synthesis of valuable chemicals. Crit Rev Biotechnol. 2024;44:163–90. 36596577 10.1080/07388551.2022.2153008

[bib123] Wang Z, Gao C, Wang Q et al. Production of pyruvate in *Saccharomyces cerevisiae* through adaptive evolution and rational cofactor metabolic engineering. Biochem Eng J. 2012;67:126–31.

[bib124] Werpy TA, Holladay JE, White JF. Top value added chemicals from biomass: I. In: Results of Screening for Potential Candidates from Sugars and Synthesis Gas: Pacific Northwest National Lab. Richland, WA: PNNL, 2004.

[bib125] Wills C . Production of yeast alcohol dehydrogenase isoenzymes by selection. Nature. 1976;261:26–29. 179008 10.1038/261026a0

[bib126] Xiao C, Pan Y, Huang M. Advances in the dynamic control of metabolic pathways in *Saccharomyces cerevisiae*. Eng Microbiol. 2023;3:100103. 39628908 10.1016/j.engmic.2023.100103 PMC11610979

[bib127] Yan D, Wang C, Zhou J et al. Construction of reductive pathway in *Saccharomyces cerevisiae* for effective succinic acid fermentation at low pH value. Bioresour Technol. 2014;156:232–9. 24508660 10.1016/j.biortech.2014.01.053

[bib128] Yang S, Madbouly SA, Schrader JA et al. Characterization and biodegradation behavior of bio-based poly(lactic acid) and soy protein blends for sustainable horticultural applications. Green Chem. 2015;17:380–93.

[bib129] Yao Z, Guo Y, Wang H et al. A highly efficient transcriptome-based biosynthesis of non-ethanol chemicals in Crabtree negative *Saccharomyces cerevisiae*. Biotechnol Biofuels. 2023;16:37. 10.1186/s13068-023-02276-5 PMC9985264 36870984

[bib130] Yu T, Liu Q, Wang X et al. Metabolic reconfiguration enables synthetic reductive metabolism in yeast. Nat Metab. 2022;4:1551–9. 36302903 10.1038/s42255-022-00654-1 PMC9684072

[bib131] Yu T, Zhou YJ, Huang M et al. Reprogramming yeast metabolism from alcoholic fermentation to lipogenesis. Cell. 2018;174:1549–1558.e14. 30100189 10.1016/j.cell.2018.07.013

[bib132] Zahoor A, Küttner FTF, Blank LM et al. Evaluation of pyruvate decarboxylase-negative *Saccharomyces cerevisiae* strains for the production of succinic acid. Eng Life Sci. 2019;19:711–20. 32624964 10.1002/elsc.201900080 PMC6999389

[bib133] Zelle RM, de Hulster E, van Winden WA et al. Malic acid production by *Saccharomyces cerevisiae*: engineering of pyruvate carboxylation, oxaloacetate reduction, and malate export. Appl Environ Microbiol. 2008;74:2766–77. 18344340 10.1128/AEM.02591-07 PMC2394876

[bib134] Zhai H, Cui L, Xiong Z et al. CRISPR-mediated protein-tagging signal amplification systems for efficient transcriptional activation and repression in *Saccharomyces cerevisiae*. Nucleic Acids Res. 2022;50:5988–6000. 35641106 10.1093/nar/gkac463 PMC9178002

[bib135] Zhang W, Kang J, Wang C et al. Effects of pyruvate decarboxylase (pdc 1, pdc 5) gene knockout on the production of metabolites in two haploid *Saccharomyces cerevisiae* strains. Prep Biochem Biotechnol. 2022a;52:62–9. 33881948 10.1080/10826068.2021.1910958

[bib136] Zhang Y, Dai Z, Krivoruchko A et al. Functional pyruvate formate lyase pathway expressed with two different electron donors in *Saccharomyces cerevisiae* at aerobic growth. FEMS Yeast Res. 2015;15:fov024. 25979691 10.1093/femsyr/fov024

[bib137] Zhang Y, Liu D, Chen Z. Production of C2-C4 diols from renewable bioresources: new metabolic pathways and metabolic engineering strategies. Biotechnol Biofuels. 2017;10:299. 29255482 10.1186/s13068-017-0992-9 PMC5727944

[bib138] Zhang Y, Liu G, Engqvist MKM et al. Adaptive mutations in sugar metabolism restore growth on glucose in a pyruvate decarboxylase negative yeast strain. Microb Cell Fact. 2015;14:116. 26253003 10.1186/s12934-015-0305-6 PMC4529725

[bib139] Zhang Y, Su M, Qin N et al. Expressing a cytosolic pyruvate dehydrogenase complex to increase free fatty acid production in *Saccharomyces cerevisiae*. Microb Cell Fact. 2020;19:226. 33302960 10.1186/s12934-020-01493-z PMC7730738

[bib140] Zhang Y, Su M, Wang Z et al. Rewiring regulation on respiro-fermentative metabolism relieved Crabtree effects in *Saccharomyces cerevisiae*. Synth Syst Biotechnol. 2022;7:1034–43. 35801089 10.1016/j.synbio.2022.06.004 PMC9241035

[bib141] Zhao EM, Zhang Y, Mehl J et al. Optogenetic regulation of engineered cellular metabolism for microbial chemical production. Nature. 2018;555:683–7. 29562237 10.1038/nature26141 PMC5876151

[bib142] Zhong W, Yang MH, Mu TZ et al. Systematically redesigning and optimizing the expression of-lactate dehydrogenase efficiently produces high-optical-purity-lactic acid in. Biochem Eng J. 2019;144:217–26.

[bib143] Zhu P, Luo R, Li Y et al. Metabolic engineering and adaptive evolution for efficient production of L-lactic acid in *Saccharomyces cerevisiae*. Microbiol Spectr. 2022;10:e0227722. 36354322 10.1128/spectrum.02277-22 PMC9769770

